# *In vitro* selection of l-DNA aptamers that bind a structured d-RNA molecule

**DOI:** 10.1093/nar/gkz1236

**Published:** 2020-01-17

**Authors:** Sougata Dey, Jonathan T Sczepanski

**Affiliations:** Department of Chemistry, Texas A&M University, College Station, TX 77843, USA

## Abstract

The development of structure-specific RNA binding reagents remains a central challenge in RNA biochemistry and drug discovery. Previously, we showed *in vitro* selection techniques could be used to evolve l-RNA aptamers that bind tightly to structured d-RNAs. However, whether similar RNA-binding properties can be achieved using aptamers composed of l-DNA, which has several practical advantages compared to l-RNA, remains unknown. Here, we report the discovery and characterization of the first l-DNA aptamers against a structured RNA molecule, precursor microRNA-155, thereby establishing the capacity of DNA and RNA molecules of the opposite handedness to form tight and specific ‘cross-chiral’ interactions with each other. l-DNA aptamers bind pre-miR-155 with low nanomolar affinity and high selectivity despite the inability of l-DNA to interact with native d-RNA via Watson–Crick base pairing. Furthermore, l-DNA aptamers inhibit Dicer-mediated processing of pre-miRNA-155. The sequence and structure of l-DNA aptamers are distinct from previously reported l-RNA aptamers against pre-miR-155, indicating that l-DNA and l-RNA interact with the same RNA sequence through unique modes of recognition. Overall, this work demonstrates that l-DNA may be pursued as an alternative to l-RNA for the generation of RNA-binding aptamers, providing a robust and practical approach for targeting structured RNAs.

## INTRODUCTION

Base-pairing properties enable RNA to fold into intricate three-dimensional structures that result in its diverse cellular functions. RNA structural motifs can influence transcription, splicing, cellular localization, stability and translation of RNA (1). Thus, RNA structure plays a role in almost every facet of gene expression. Furthermore, structured RNA elements have been discovered to play critical roles in a variety of diseases, including viral infections, neurological disorders and cancer (2–4). Given the importance of RNA in human health and disease, targeting RNAs based on their unique structures is a potentially transformative diagnostic and therapeutic strategy. Furthermore, structure-specific reagents open the door to develop novel tools to study RNA function. However, discovery of molecules that are capable of binding RNA structures with high affinity and specificity has proven challenging using current approaches. For example, RNAs with extensive secondary structures are often inaccessible to traditional hybridization-based reagents (5,6). Furthermore, RNA-binding small molecules often suffer from poor selectivity due to RNA’s negatively charged backbone and structural redundancy (7–9). Therefore, development of structure-specific RNA binding reagents remains a central challenge.

A potential solution to this problem is the use of aptamers (10,11), which are short single-stranded nucleic acid molecules capable of binding to a specific target. In theory, it should be possible to evolve an aptamer against any RNA target using *in vitro* selection methods. Indeed, several groups have successfully isolated aptamers capable of binding structured RNA molecules (12–16). More recently, we demonstrated that aptamers composed of l-ribose-based nucleic acids (l-RNA), which are the synthetic enantiomers of natural d-nucleotides, can also be evolved to bind tightly to RNA structures, and may be particularly well suited for this purpose (17–19). This is because d- and l-oligonucleotides are incapable of forming contiguous WC base pairs with each other (20–22), instead forcing l-aptamers to interact with native d-RNA through tertiary interactions. This unique mode of nucleic acid recognition, referred to as ‘cross-chiral’ recognition, is expected to be more specific than simple WC pairing as it depends on both the sequence *and* structure (i.e. shape) of the target RNA. Despite the inability of l-aptamers to form WC base pairs with RNA, high-affinity binding can still be achieved. For example, we previously isolated a cross-chiral l-RNA aptamer capable of binding the hairpin structure of precursor microRNA (pre-miR)-155 with a *K*_d_ of 11 nM, making it one of the tightest binding, non-hybridization-based reagents ever developed against a structured RNA target (18). This aptamer was also shown to inhibit Dicer-mediated processing of pre-miR-155 *in vitro*. Therefore, in addition to high affinity and selectivity, cross-chiral aptamers are capable of modulating RNA function by blocking RNA–protein interactions. Importantly, because l-oligonucleotides are orthogonal to the stereospecific environment of native biology, cross-chiral aptamers are completely resistant to nuclease degradation (22,23) and expected to be less susceptible to off-target interactions with native biomacromolecules, including other nucleic acids. Taken together, these properties suggest that cross-chiral aptamers hold great promise as structure-specific RNA affinity reagents, especially for application in biological environments.

All cross-chiral aptamers reported to date have been composed of l-RNA. Despite their attractive properties, however, the use of l-RNA places potential limits on the practical utility of these aptamers. In particular, the inefficiency of solid-phase RNA synthesis and the relative high cost of l-nucleoside phosphoramidites, makes it difficult to obtain the needed quantity of l-RNA cross-chiral aptamers for many downstream applications. In addition, although l-RNA is nuclease resistant, it is still vulnerable to hydrolytic cleavage of the sugar-phosphate backbone due to the presence of the 2′-OH group. This chemical instability may prevent the use of cross-chiral l-RNA aptamers within certain biological/chemical environments and limit their ability to be stored long-term, especially in low-resource settings. In contrast, the use of l-DNA may offer a more practical approach. Compared to RNA, solid-phase synthesis of DNA oligonucleotides is more rapid, higher yielding and does not require arduous 2′-OH deprotection procedures. As a result, the use of a DNA backbone is expected to greatly simplify the post-selection synthesis of cross-chiral l-aptamers, while substantially increasing the amount of material that can be obtained. Furthermore, the lack of a 2′-OH group makes l-DNA considerably more stable than l-RNA, and thus, compatible with a more diverse range of environments.

While both DNA and RNA libraries (and more recently XNA libraries) have yielded high-affinity aptamers against a diverse range of targets (24), and in some cases the same target, cross-chiral recognition between DNA and RNA cannot be assumed on this basis. Early work into d/l-hybrids showed that RNA had a greater propensity for binding the enantiomer of its complement than did DNA, although these interactions did not take place through WC base pairing (22,25). The conformational rigidity of the sugar backbone has also been found to correlate positively with formation of stable heterochiral complexes (26). Furthermore, in the absence of typical WC base pairing between l-aptamers and d-RNA, the 2′-OH group present on l-RNA aptamers may provide critical interactions needed to form stable cross-chiral complexes.

With the above considerations in mind, the goal of the current study was to determine whether cross-chiral aptamers could be isolated from libraries of DNA rather than RNA, and if so, can similar RNA-binding properties be achieved. Using mirror-image *in vitro* selection methods, we generated several l-DNA aptamers against the hairpin structure of pre-miR-155, a prototypical oncogenic microRNA with clinical implications. A parallel selection experiment was also carried out employing the cationic nucleotide 5-aminoallyl-2′-deoxyuridine (5AdU), yielding cross-chiral l-DNA aptamers harbouring the same modification. Both modified and unmodified l-DNA aptamers bound pre-miR-155 with low nanomolar affinity, similar to what previously reported for l-RNA aptamers for the same target. In the case of the modified l-DNA aptamer, the cationic moiety was critical for binding. The sequence and structure of cross-chiral l-DNA aptamers isolated herein are distinct from their RNA counterparts despite binding to the same site (i.e. nucleotide sequence) on the target RNA. At last, we showed that formation of l-aptamer–pre-miR-155 complexes inhibit Dicer cleavage *in vitro*, thereby blocking formation of the mature miR-155. Overall, our results establish the capacity of l-DNA aptamers to bind native d-RNA structures (and *vice versa*), and show that the absence of the 2′-hydroxyl group is not a disadvantage for cross-chiral nucleic acid recognition.

## MATERIALS AND METHODS

### Materials

Oligonucleotides were either purchased from Integrated DNA Technologies (Coralville, IA, USA) or prepared by solid-phase synthesis on an Expedite 8909 DNA/RNA synthesizer. Synthesizer reagents and d-nucleoside phosphoramidites were purchased from Glen Research (Sterling, VA, USA). d-5-aminoallyl-dUTP was purchased from TriLink Biotechnologies (San Diego, CA, USA). l-nucleoside phosphoramidites were purchased from ChemGenes (Wilmington, MA, USA). All oligonucleotides were purified by polyacrylamide gel electrophoresis (PAGE) and desalted by ethanol precipitation. Histidine-tagged KOD mutant (*exo-*) polymerase was expressed and purified as previously reported (27). Streptavidin coated magnetic beads (MyOne Streptavidin C1 Dynabeads) were purchased from Life Technologies (Carlsbad, CA, USA) and high capacity streptavidin agarose beads (50% slurry) were purchased from Thermo Fischer Scientific (Rockford, IL, USA). [γ-^32^P]ATP was purchased from Perkin Elmer (Waltham, MA, USA) and dNTPs were purchased from Sigma Aldrich (St Louis, MO, USA). Mass spectrometry analysis of purified oligonucleotides was performed by Novatia, LLC (Newtown, PA, USA). All the other chemicals were purchased from either Sigma-Aldrich (St Louis, MO, USA) or Alfa Aesar (Haverhill, VA, USA) and used without further purification.

### Methods

Preparation of the l-5-aminoallyldeoxyuridine phosphoramidites (l-5AdU-CEP) and aptamer l-AdU5t is described in detail in the ‘Supplementary Methods’ section.

### Preparation of ssDNA libraries

A single-stranded DNA (ssDNA) library (Lib.86) containing a 45 nt random domain flanked by two primer binding regions was amplified in a 10 ml polymerase chain reaction (PCR) reaction containing 0.5 μM of each primers Fwd.86 and Rev.86, 50 mM KCl, 1.5 mM MgCl_2_, 0.1% TRITON-X, 10 mM Tris (pH 9.0) and 0.5 mM each of the four dNTPs (see Supplementary Table S1 for sequences). The dsDNA product was ethanol precipitated, redissolved in 500 μl water and mixed with 1.5 ml of a ∼20% slurry of high-capacity streptavidin coated agarose beads in Buffer WB (50 mM NaCl, 10 mM Tris, pH 7.6). The bead-binding reaction was allowed to occur for 30 min at room temperature with shaking and complete immobilization of the DNA was confirmed by agarose gel electrophoresis. The supernatant was removed via filtration, and the beads were washed two times with 500 μl Buffer WB and once with water. The non-biotinylated strand (Lib.dT) was then eluted from the beads using 200 μl ice cold Buffer EB (50 mM NaOH, 1 mM ethylenediaminetetraacetic acid (EDTA)) and the eluent immediately neutralized with 20 μl of Buffer NB1 (1 M Tris, pH 7.6 and 3 M NaOAc) and ethanol precipitated. The beads were washed three times with 1 ml Buffer WB and resuspended in a reaction mixture (3.6 ml) consisting of 4 μM primer Fwd.86, 500 μM each dNTP (dATP, dCTP, dGTP and 5-aminoallyl-dUTP), 36 U KOD Dash DNA Polymerase (40:1 mixture of KOD mutant *exo-* and KOD DNA polymerase) (27), 10 mM KCl, 15 mM MgSO_4_, 6 mM (NH_4_)_2_SO_4_, 0.01% bovine serum albumin (BSA), 0.01% TRITON-X and 120 mM Tris, pH 8.0. The reaction was then heated to 95°C for 1 min, annealed at 46°C for 30 s and incubated at 70°C for 30 min. The supernatant was removed by filtration and the beads washed two times with 500 μl Buffer WB. The modified ssDNA strand (Lib.AdU) was eluted from the beads using 200 μl ice cold Buffer EB and the eluent immediately neutralized with 20 μl of Buffer NB1 and ethanol precipitated. The beads were quickly washed three times 1 ml Buffer WB and the above extension reaction repeated. Overall, three extension reactions were carried out on the same portion of beads. All eluted DNA libraries were purified by denaturing PAGE (10%, 19:1 acrylamide:bis-acrylamide) prior to use.

### *In vitro* selection

Separate *in vitro* selection experiments were carried out for Lib.dT and Lib.5AdU. A 500 μl reaction mixture containing 1 nmol of ssDNA library (∼10^14^ unique sequences) in Buffer SB (5 mM MgCl_2_, 50 mM KCl, 20 mM NaCl, 25 mM Tris, pH 7.6) was heated at 90°C for 3 min and allowed to cool slowly to room temperature. The reaction mixture was incubated with 100 μl of streptavidin-coated Dynabeads (previously blocked with 1 mg/ml yeast tRNA in Buffer SB) and incubated at room temperature for 1 h. The supernatant was removed and the beads discarded to remove any bead-binding oligonucleotides (i.e negative selection step). To the retained supernatant was added 100 pmol of l-pre-miR-155t and the solution was incubated at room temperature for 30 min. The reaction mixture was then mixed with 100 μl streptavidin-coated Dynabeads and incubated at room temperature for an additional 15 min. The beads were then washed five times with 1 mL Buffer SB in order to remove weakly bound ssDNA molecules and the retained ssDNA was eluted using 300 μl Buffer EB. The elute ssDNA was quickly neutralized with a solution containing 30 μl 3 M NaOAc, 30 μl of 1 M Tris (pH 7.6) and 4 μl glycogen (1 mg/ml, Roche Diagnostics, Indianapolis, IN, USA). The DNA was then ethanol precipitated and used directly in a 100 μl PCR amplification reaction containing 1 U KOD Dash DNA polymerase, 0.5 μM primer Fwd.86, 0.5 μM primer Rev.86, 120 mM Tris, pH 8.0, 10 mM KCl, 15 mM MgSO_4_, 6 mM (NH_4_)_2_SO_4_, 0.01% BSA, 0.01% TRITON-X and 0.5 mM each of the four dNTPs. For the first cycle of amplification, the reaction mixture was heated to 95°C for 2 min, annealed at 46°C for 30 s and extended at 70°C for 30 min. All subsequence cycles were carried out using the following program: 95°C for 30 s, 46°C for 30 s and 70°C for 1 min. The amplified DNA was ethanol precipitated and used to generate a new DNA library for the next round of *in vitro* selection. Supplementary Figure S2 summarizes selection conditions for each round. Following round 8, the enriched dsDNA pool was cloned into *Escherichia coli* using the TOPO TA cloning kit (Life Technologies, Carlsbad, CA, USA). Bacteria were grown for 16 h at 37°C on LB agar plates containing 50 μg/mL kanamycin. Individual colonies were amplified by PCR and sequenced by Eton Biosciences Inc. (San Diego, CA, USA).

### Transcription of RNA

DNA templates used to prepare the various versions of d-pre-miR-155 were generated by cross-extension of two overlapping synthetic oligonucleotides (Supplementary Table S6). The oligonucleotides (2 nmol each) where annealed in a 500 μl reaction mixture containing 6 mM MgCl_2_, 150 mM KCl, 20 mM DTT and 100 mM Tris (pH 8.3), which was heated at 90°C for 1 min and then cooled slowly to room temperature. Reverse Transcriptase (16 U/μl) was added and the reaction incubated at 42°C for 45 min. Following ethanol precipitation, the resulting dsDNA was added to a transcription reaction mixture containing 10 U/μl T7 RNA polymerase, 0.001 U/μl inorganic pyrophosphatase (IPP), 25 mM MgCl_2_, 2 mM spermidine, 10 mM DTT, 40 mM Tris pH 7.9 and 5 mM of each of the four NTPs. After incubating for 2 h at 37°C, reaction mixture was ethanol precipitated and the RNA purified by denaturing PAGE (10%, 19:1 acrylamide:bis-acrylamide).

### Electrophoretic mobility shift assay

Dissociation constants for aptamer–pre-miR complexes were determined as described previously (18). Briefly, either 5 nM of 5′-fluoroscein (FAM)-labeled l-pre-miR-155t or 1 nM 5′-[^32^P]-labeled d-pre-miR-155(t) was incubated with various concentrations (0–3000 nM) of the indicated DNA aptamer in a 20 μl reaction mixture containing either 2 or 5 mM MgCl_2_, 50 mM KCl, 20 mM NaCl, 0.01 mg/ml tRNA and 25 mM Tris (pH 7.6). After 30 min, the samples were analysed by non-denaturing PAGE (8%, 19:1 acrylamide:bis-acrylamide), which contained either 2 or 5 mM MgCl_2_, 50 mM KOAc, 20 mM NaOAc and 25 mM Tris (pH 7.6). Electrophoresis was carried out at 30 mA for ∼3 h. The gel was imaged by either autoradiography or by fluorescence emission using a Typhoon FLA-9500 Molecular Imager (General Electric Co., Boston, MA, USA).

### In-line probing analysis of d-pre-miR-155t

A 20 μl reaction mixture containing 100 nM of 5′-[^32^P]-labeled d-premiR-155t, either 0 or 20 μM l-aptamer, 5 mM MgCl_2_, 50 mM KCl, 20 mM NaCl and 25 mM Tris (pH 7.6) was incubated at room temperature for 48 h. The cleavage products were resolved by denaturing PAGE (15%, 19:1 acrylamide:bis-acrylamide) and imaged by autoradiography using a Typhoon FLA-9500 Molecular Imager.

### Circular dichroism (CD) spectroscopy

The indicated DNA aptamer (9 μM) in Buffer CD (20 mM NaCl, 25 mM Tris, pH 7.6) supplemented with 50 mM of either KCl or LiCl was folded by heating at 90°C for 3 min before being cooled slowly to room temperature. Data were obtained from a 450 μl sample in a quartz cuvette using an Applied Photophysics Chirascan spectrophotometer (Leatherhead, England) at 1 nm intervals with time per point 1.5 s from 220 to 310 nm. All data were collected at a constant temperature of 24°C.

### DMS footprinting

Dimethyl sulphate (DMS) footprinting was carried out according to the protocol described by Laurence *et al.* (28). Briefly, 1 μM of the indicated [^32^P]-labeled d-DNA aptamer was refolded by heating at 90°C for 5 min and then cooling slowly to room temperature in reaction mixture (20 μl) containing 5 mM MgCl_2_, 20 mM NaCl, either 50 mM KCl or 50 mM LiCl, and 25 mM Tris (pH 7.6). For each aptamer, a parallel reaction mixture (20 μl) was prepared that also contained 1 μM l-pre-miR-155t. Each reaction mixture was treated with 2 μl DMS (10% dimethyl sulphate by volume in 1:1 ethanol:water), mixed well and allowed to react for 2 min at room temperature. The reaction was quenched with 80 μl of a solution containing 0.5 M NaOAc (pH 5.6) and 1 mg/ml tRNA, and ethanol precipitated. The DNA was pelleted by centrifugation and subsequently dissolved in 100 μl of a 1 M piperidine solution (in water). After heating at 90°C for 30 min, the solution was cooled to room temperature and concentrated by vacuum. The resulting pellets were dissolved in Buffer LB (4 M urea, 10 mM EDTA) and resolved by denaturing PAGE (15%, 19:1 acrylamide:bis-acrylamide). The gel was imaged by autoradiography using a Typhoon FLA-9500 Molecular Imager.

### Dicer inhibition assay

The indicated concentration of l-DNA aptamer was added to a reaction mixture (20 μl) containing 1 nM 5′-[^32^P]-labeled d-pre-miR-155, 5 mM MgCl_2_, 20 mM NaCl, 50 mM KCl, 0.01 mg/ml tRNA, 10 mM DTT and 25 mM Tris (pH 7.6), and the reaction was initiated by the addition of 10 nM human Dicer protein. After 30 min, an aliquot was removed and quenched with a two volumes of formamide loading buffer (95% formamide, 10 mM EDTA) and the cleavage products were resolved by denaturing PAGE (10%, 19:1 acrylamide:bis-acrylamide). The gel was imaged by autoradiography using a Typhoon FLA-9500 Molecular Imager.

## RESULTS AND DISCUSSION

### Discovery of cross-chiral DNA aptamers for pre-miR-155

**Figure 1.**
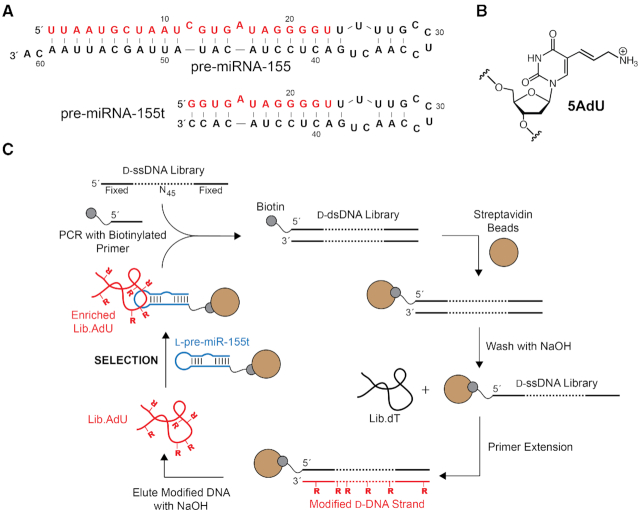
Mirror image *in vitro* selection of cross-chiral DNA aptamers for pre-miR-155. (**A**) Sequences and secondary structures of pre-miR-155 and truncated variant pre-miR-155t. For consistency, numbering of nucleotides is maintained upon truncation. (**B**) Chemical structure of 5-aminoallyl-2′-deoxyuridine (5AdU). (**C**) Schematic representation of the *in vitro* selection method used to isolate modified DNA aptamers. For unmodified aptamers, Lib.dT is directly applied to the selection step.

For this study, we chose precursor microRNA-155 (pre-miR-155) as the model target (Figure 1A). The mature form of pre-miR-155, microRNA-155 (miR-155), is a prototypical oncogenic miR whose overexpression has been linked with the development and invasiveness of several human cancers, making it an attractive therapeutic target (29). Furthermore, two l-RNA aptamers were previously selected against pre-miR-155 (18), suggesting that this hairpin structure favors cross-chiral recognition. These l-RNA aptamers also serve as a benchmark for evaluating the cross-chiral binding properties of l-DNA. l-Aptamers are initially selected as d-aptamers against the enantiomer of the target ligand, which enables enzymatic amplification of the d-oligonucleotides during the process of *in vitro* selection. Therefore, we chemically synthesized a 38 nt truncated version of pre-miR-155 (l-pre-miR-155t) using commercially available l-nucleoside phosphoramidites (Figure 1A). l-pre-miR-155t was biotinylated at its 3′ end allowing it to be immobilized on streptavidin-coated beads during the selection step.

We prepared two single-stranded d-DNA libraries: one comprised of canonical deoxynucleotides (Lib.dT) and one containing 5AdU in place of dT (Lib.AdU) (Figure 1B). We hypothesize that merging the shape-based recognition of l-aptamers with functional groups absent in natural nucleic acids will generate RNA-binding properties that are unachievable using current modalities. Indeed, the use of modified nucleotides is a well-proven strategy for increasing the success rate of *in vitro* selection experiments against more demanding targets, and has been shown to facilitate evolution of d-aptamers with significantly improved activities over their unmodified counterparts (30–32). Importantly, incorporation of cationic modifications into selection libraries has previously led to identification of aptamers that bind tightly to negatively charged ligands, including RNA (17,33–35). The 5AdU-containing DNA library was generated using a modified version of the procedure originally reported by Eaton *et al.* (Figure 1C) (36). Briefly, a ssDNA library (∼10^14^ unique sequences) containing a 45 nt random domain flanked by fixed primer binding sites (Supplementary Table S1) was amplified by PCR in order to generate multiple copies of each DNA molecule. One of the primers used during PCR was biotinylated at its 5′ end, allowing for the immobilization of the double-stranded product on streptavidin coated agarose beads following amplification. The non-biotinylated strand was subsequently removed via NaOH treatment (0.1 M) and became the starting pool for the unmodified *in vitro* selection experiment (i.e. Lib.dT). The bead-bound DNA strand was then used as a template for a primer extension reaction employing 5-aminoallyl-dUTP in place of dTTP. The extension was carried out using KOD Dash DNA polymerase, which was previously shown to be highly tolerant of dUTP derivatives modified at the C5 position (36). Following the extension step, the newly generated 5AdU-modified DNA strand was separated from the bead-bound template via NaOH treatment and further purified by gel electrophoresis. Multiple extension reactions were typically carried out on the same portion of template-bound beads in order to generate a sufficient quantity of Lib.AdU for the selection experiment (Supplementary Figure S1).

Separate *in-vitro* selection experiments were carried out for each of the two libraries (Lib.T and Lib.AdU) (Figure 1C). Each d-DNA library was incubated together with the biotinylated l-pre-miR-155t target in a reaction mixture containing 5 mM MgCl_2_, 20 mM NaCl, 50 mM KCl, 0.1%TWEEN-20 and 25 mM Tris (pH 7.6) at room temperature. d-DNA molecules that bound l-pre-miR-155t were captured using streptavidin-coated magnetic beads, which were subsequently washed with the same binding solution. Aptamers that remained bound to the beads were eluted using NaOH (0.1 M) and amplified by PCR using KOD Dash DNA polymerase. As before, a biotinylated primer was used during the amplification step and the resulting double-stranded PCR product was used to generate the corresponding pool of single-stranded DNA for the next round of *in vitro* selection.

A total eight rounds of *in vitro* selections were carried out for both unmodified (Lib.dT) and modified (Lib.AdU) d-DNA libraries. The selection pressure was increased during successive rounds by reducing the concentration of the d-DNA library from 2000 to 100 nm, and by increasing the duration of the bead-washing steps (see Supplementary Figure S2 for details). The temperature at which the selection was carried out was also raised from 23°C to 37°C during the final two rounds. Following the final round of *in vitro* selection, the enriched Lib.T and Lib.AdU libraries were cloned and sequenced (Supplementary Tables S2 and 3, respectively).

**Figure 2.**
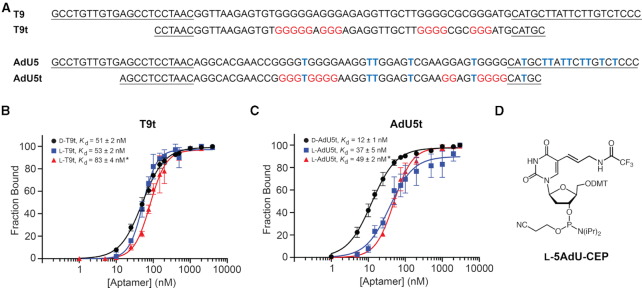
Sequence and binding affinity of cross-chiral DNA aptamers. (**A**) Sequences of full-length aptamers T9 and AdU5 and their truncated variants. Blue Ts indicate the position of 5AdU and primer-binding sites are underlined. Guanosine residues shown to be involved in G-quadruplex formation are colored red (see Figure 4). (**B**) Saturation plots for binding of T9t to either pre-miR-155t or pre-miR-155 (asterisk) having the opposite chirality. (**C**) Saturation plots for binding of AdU5t to either pre-miR-155t or pre-miR-155 (asterisk) having the opposite chirality. *K*_d_ values reported as mean ± S.D. (*n* = 3). (**D**) Chemical structure of 5-aminoallyl-dU phosphoramidite (**l-5AdU-CEP**).

Five clones from both the unmodified (Lib.T) and modified (Lib.AdU) DNA libraries, each representing a unique aptamer sequence, were screened for binding l-pre-miR-155 using an electrophoretic mobility shift assay (EMSA) (Supplementary Figure S3). Interestingly, one sequence (d-AdU2 and d-T1) appeared within both enriched populations and was capable of binding l-pre-miR-155t regardless of its modification state. Nevertheless, clones d-T9 and d-AdU5 from Lib.T and Lib.AdU, respectively, emerged as the tightest binders, and thus, all further studies were based on these two sequences (Figure 2A). d-T9 and d-AdU5 bound l-pre-miR-155t with *K*_d_ values of 60 ± 4 and 36 ± 2 nM, respectively. To the best of our knowledge, these aptamers represent the first example of a stable interaction between DNA and RNA of the opposite chirality. Importantly, the *K*_d_ values for d-T9 and d-AdU5 were similar to the previously reported RNA aptamer for the same target (*K*_d_ = 19 nM) (18), suggesting that the absence of the 2′-hydroxyl group is not a disadvantage for cross-chiral nucleic acid recognition. A version of d-AdU5 containing dT rather than 5AdU was unable to bind l-pre-miR-155t, demonstrating that binding is dependent on the cationic modification (Supplementary Figure S4a). Similarly, replacing dT with 5AdU within the unmodified d-T9 aptamer significantly impaired its ability to bind l-pre-miR-155t. This observation shows that simply appending a cationic moiety to an aptamer does not itself improve binding, but rather precise positioning of the modification is required and must be achieved through *in vitro* selection.

### Preparation of truncated l-DNA aptamers with affinity for d-pre-miR-155

We next sought to determine the minimal binding domain for aptamers d-T9 and d-AdU5, both of which were >80 nt in length. Our primary goal was to identify truncated sequences that could be prepared efficiently by chemical oligonucleotide synthesis using l-nucleoside phosphoramidites. The minimal binding domains of d-T9 and d-AdU5 were determined by generating variants with a succession of 5′ and 3′ truncations and screening for binding by EMSA (Supplementary Tables S4 and 5). This iterative process resulted in the reduction of d-T9 into a 56 nt sequence (d-T9t) and d-AdU5 into a 60 nt sequence (d-AdU5t) having *K*_d_ values of 51 ± 2 and 12 ± 1 nM, respectively, when measured under the *in vitro* selection conditions (i.e. 5 mM Mg^2+^) (Figure 2B and C). It was fortuitous, but not uncommon, that both truncated aptamers showed higher binding affinity to pre-miR-155t than either full-length parent aptamer. Scrambled versions of both truncated d-aptamers failed to bind l-pre-miR-155 (Supplementary Figure S4b). It is worth noting that truncation of aptamer d-AdU5 reduced the number of 5AdU residues from 14 to 6 (Figure 2A), indicating that the majority of cationic modifications within d-AdU5 were dispensable for binding l-pre-miR-155.

Next, we prepared l-DNA versions of both truncated aptamers via solid-phase DNA synthesis using l-nucleoside phosphoramidites (Supplementary Figures S5 and 6). The 5AdU modification was incorporated into l-AdU5t using l-5AdU-CEP (Figure 2D), which was synthesized according to published procedures (see Supplementary Data) (37–39). In general, a standard 1 μM scale synthesis yielded ∼100 nmol of purified l-DNA aptamer. This is compared to ∼10–30 nmols for the previously reported l-RNA aptamer against pre-miR-155 (data not shown). As is dictated by the principal of reciprocal chiral substrate specificity (40), the affinity of both l-DNA aptamers for d-pre-miR-155t was very similar to their corresponding d-DNA aptamers for l-pre-miR-155t (Figure 2B and C). l-T9t and l-AdU5t bound d-pre-miR-155t with *K*_d_ values of 53 ± 2 and 37 ± 5 nM, respectively, when measured under *in vitro* selections conditions. The somewhat lower affinity of l-AdU5t relative to d-AdU5t (∼3-fold) likely reflects the poorer quality of synthetic l-DNA compared to d-DNA generated by enzymatic polymerization, as evident by mass spectrometry (Supplementary Figure S6). For example, acrylonitrile adducts were observed on l-AdU5t but not d-AdU5t, which are a result of the deprotection step during solid-phase oligonucleotide synthesis. The affinity of l-T9t and l-AdU5t for d-pre-miR-155t decreased ∼3- to 4-fold when the concentration of Mg^2+^ in the binding buffer was reduced from 5 to 2 mM, indicating that both aptamers were dependent on Mg^2+^ (Supplementary Figure S7). Importantly, both l-DNA aptamers bound the full-length d-pre-miR-155 RNA hairpin (which is the intended target) with similar *K*_d_ values as the truncated target used during *in vitro* selection (Figure 2B and C). Not surprisingly, neither T9t nor AdU5t aptamers were able to bind pre-miR-155 of the same chirality (Supplementary Figure S8).

### Aptamers T9t and AdU5t form G-quadruplex structures

**Figure 3.**
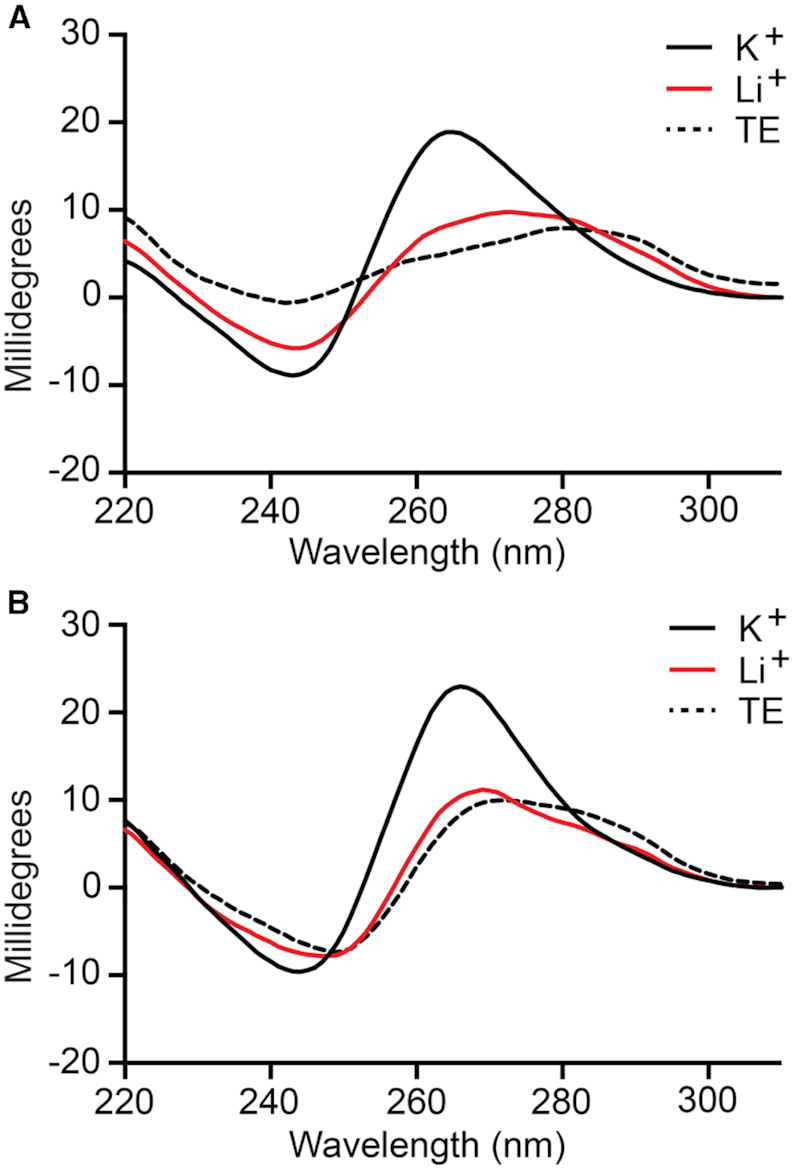
CD spectrum of d-T9t (**A**) and d-AdU5t (**B**) aptamers. All CD spectrum were obtained with 9 μM aptamer in the presence of a buffer containing 20 mM NaCl, 25 mM Tris (pH 7.6) and either 50 mM KCl (K^+^, shown in black solid line) or LiCl (Li^+^, shown in red solid line) at 23°C. CD spectra recorded under no salt condition were prepared in TE buffer (shown in dashed black line).

The DNA structure prediction software Mfold (41) failed to identify stable secondary structures within T9t and AdU5t aptamer sequences (Supplementary Figure S9). However, both minimal aptamers contained at least four clusters of consecutive guanine residues that are indicative of G-quadruplexes (Figure 2A), which is not uncommon for DNA aptamers (42). Therefore, we wished to determine whether T9t and AdU5t form G-quadruplex structures and if such structures are important for binding pre-miR-155. In the absence of K^+^, which is known to stabilize G-quadruplexes more effectively than other monovalent ions (43), both d-DNA aptamers failed to bind l-pre-mir-155t, even at concentrations of aptamer far exceeding the observed *K*_d_ (Supplementary Figure S10a). The CD spectrum of d-T9t and d-AdU5t in the presence of ion concentrations used during the *in vitro* selection experiment (20 mM Na^+^ and 50 mM K^+^) showed a negative peak at ∼240 and a positive peak at ∼260 nm (Figure 3), which are characteristic of a parallel-stranded G-quadruplex structure (44,45). Replacing K^+^ with Li^+^, which does not facilitate G-quadruplex formation (43), resulted in the loss of these features (Figure 3), instead giving CD spectrum that more closely resembled that of d-T9t and d-AdU5t obtained in the absence of any ions (i.e. TE buffer). As expected, similar results were obtained using the l-DNA versions of both aptamers, except that their CD spectrum were inverted (Supplementary Figure S10b and c) (46). Taken together, these data strongly suggest that T9t and AdU5t aptamers contain a potassium-dependent, parallel-stranded G-quadruplex structure, the formation of which is required for binding to pre-miR-155.

**Figure 4.**
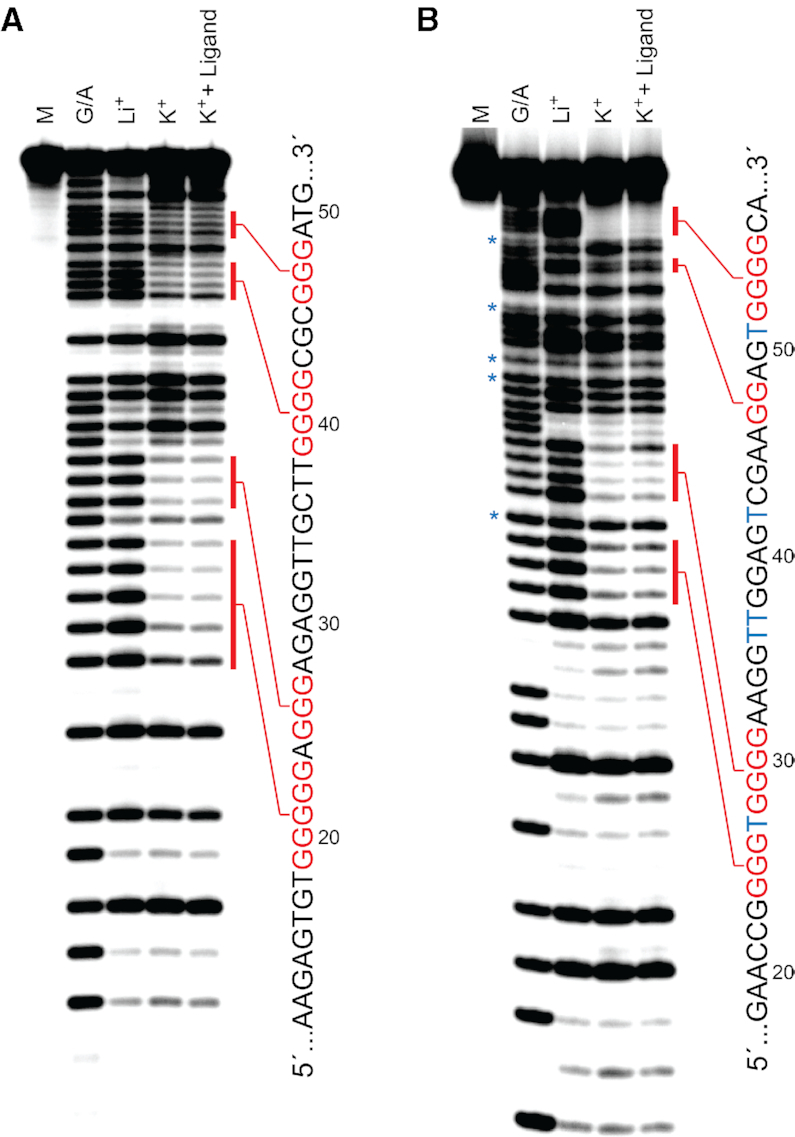
Characterization of G-quadruplex formation in d-T9t (**A**) and d-AdU5t (**B**) aptamers. Each aptamer was annealed in the presence of LiCl, KCl or KCl plus l-pre-miR-155t and subjected to DMS footprinting. The nucleotide sequence of each aptamer is shown to the right of the gel. Protected guanosine residues are colored red and 5AdU is represented by blue Ts. Treatment of d-AdU5t with formic acid (G/A) and DMS resulted in cleavage at 5AdU residues (asterisks). M = untreated control.

In order to confirm the above results and gain additional information regarding the guanine residues involved in G-quadruplex formation, we carried out a dimethyl sulphate (DMS) footprinting assay (28). The availability of guanine N7 for methylation by DMS in single-stranded and duplex DNA, but not in G-quadruplexes, is a diagnostic indicator of G-quadruplex structures. The DMS cleavage patterns of both d-T9t and d-AdU5t revealed four G-rich tracts that were strongly protected from cleavage in the presence of K^+^, implying that these residues are indeed involved in formation of a G-quadruplex (Figure 4). Interestingly, for both aptamers, the number of protected guanines varied between G-tracks. For example, d-T9t had two tracks of three guanines (G25–G27 and G46–48), one track of four guanines (G39–G42), and one track of five guanines (G19–G23) that were protected from DMS cleavage (Figure 4A). This observation is consistent with the presence of redundant guanines within the longer G-tracks, and indicates that the observed DMS cleavage patterns represent a mixture of several G-quadruplex structures each consisting of a different configuration of guanine residues (47). In the future, it will be interesting to determine whether eliminating this degeneracy leads to improved binding properties. G-quadruplex formation was not dependent on ligand binding, since inclusion of l-pre-miR-155t during the DMS assay did not significantly alter the cleavage pattern for either aptamer. This also suggests that the protected guanine bases are unlikely to be involved in hydrogen bonding to pre-miR-155. Interestingly, a previously identified cross-chiral RNA aptamer for pre-miR-155 (aptamiR-155.2) does not contain a G-quadruplex, as evident by the lack of monovalent ion dependency in its CD spectrum (Supplementary Figure S10d). Thus, despite binding to the same RNA target (i.e. the same sequence of nucleotides), DNA aptamers T9t and AdU5t form entirely different structural motifs compared to their RNA counterpart. In addition to T9t and AdU5t, several other DNA aptamer sequences identified following *in vitro* selection have at least four clusters of consecutive guanine residues that are indicative of G-quadruplexes (Supplementary Tables S2 and 3), suggesting that this could be a common structural motif for cross-chiral recognition between DNA and RNA. It may also indicate that G-quadruplexes provide a stable structural scaffold when divalent cations (e.g. magnesium) are limited. It will be interesting to select l-DNA aptamers against a diverse range of RNA structures in order to determine whether G-quadruplex formation is a general phenomenon.

### l-DNA aptamers bind selectively to the distal loop of pre-miR-155

**Figure 5.**
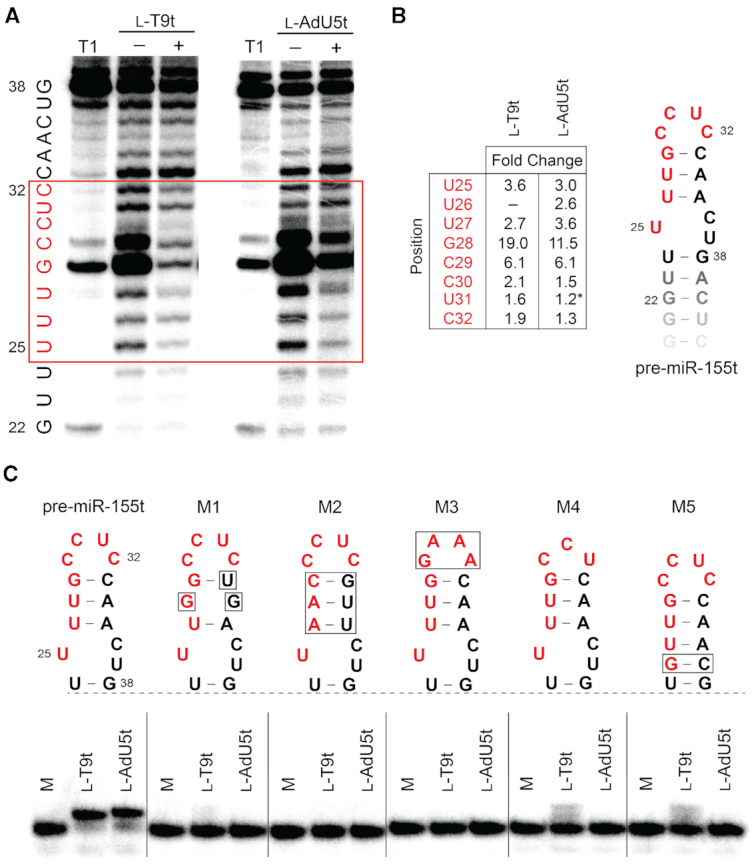
Cross-chiral DNA aptamers bind selectively to the loop domain of pre-miR-155. (**A**) In-line probing analysis of pre-miR-155t in the presence and absence of the indicated l-aptamer. Residues that underwent significant conformational changes (i.e. protection from hydrolysis) in the presence of excess l-aptamer are boxed in red. Partial digestion by ribonuclease T1 (cleavage after G residues) is shown to the left (T1). Uncropped gel images are shown in Supplementary Figures S11a and b. (**B**) The extent of pre-miR-155 protection differs between aptamers. Fold change was determined by dividing the fraction cleaved in the absence of the aptamer by that in its presence (i.e. greater fold change corresponds to greater protection). Asterisk indicates increased hydrolysis in the presence of the aptamer. (**C**) Binding of excess l-T9t and l-AdU5t to 5′-[^32^P]-pre-miR-155t or modified variants. All binding reactions contain 3 μM aptamer, 1 nM 5′-[^32^P]-pre-miR-155t (or variant), 5 mM MgCl_2_, 50 mM KCl, 20 mM NaCl, and 25 mM Tris (pH 7.6). Variant M1 is mouse pre-miR-155. M = 5′-[^32^P]-pre-miR-155t (or variant) control.

The l-pre-miR-155t target used during *in vitro* selection was truncated relative to the native pre-miR-155 RNA. This was done intentionally in order to encourage isolation of aptamers capable of binding to the loop domain, which has been shown to inhibit Dicer-mediated miR biogenesis *in vitro* (17,18). In order to determine whether l-T9t and l-AdU5t bound the loop of pre-miR-155, we performed an in-line hydrolysis analysis of pre-miR-155t in the presence and absence of saturating concentrations of l-DNA aptamer (Figure 5A) (48). Uncatalyzed in-line hydrolysis (i.e. cleavage) of phosphodiester linkages in RNA requires an in-line geometry (180°) between the 2′-oxygen nucleophile, the phosphorus electrophile and 5′-oxygen leaving group. Binding of the l-aptamer to d-pre-miR-155t is expected to restrict the conformational freedom of residues at the binding site, thereby hindering their ability to achieve the in-line geometry needed for cleavage. Incubating d-pre-miR-155t with either l-T9t or l-AdU5t led to the protection of the same 8-nucleotide region from cleavage (U_25_–C_32_), strongly suggesting that both l-aptamers bound to this site within the loop domain (Figure 5A). Interestingly, previously reported l-RNA aptamers for pre-miR-155 also bound these same residues, suggesting that this binding sight is particularly well suited for cross-chiral recognition (18). Despite their similar footprints, however, the extent to which l-T9t and l-AdU5t protected d-pre-miR-155t from hydrolysis were distinct (e.g. residue G28; Figure 5B). Because the rate of in-line hydrolysis is highly dependent on linkage geometry, which is dictated by the overall tertiary structure of the RNA, these distinct cleavage patterns likely represent two unique heterochiral interfaces that perturb the structure of pre-miR-155 in different ways. This observation is consistent with the sequence and structural differences between T9t and AdU5t. In order to validate the in-line hydrolysis data, we also showed that both aptamers failed to bind several versions of d-pre-miR-155t containing mutations and/or deletions that perturbed the structure of the loop domain (Figure 5C). Both aptamers also failed to bind several other pre-miR hairpin structures, including the mouse homologue of pre-miR-155 (M1) (Supplementary Figure S11c). Together, the above results indicate that l-T9t and l-AdU5t are selective for binding the loop domain of pre-miR-155.

### l-DNA cross-chiral aptamers inhibit Dicer-mediated cleavage of pre-miR-155

**Figure 6.**
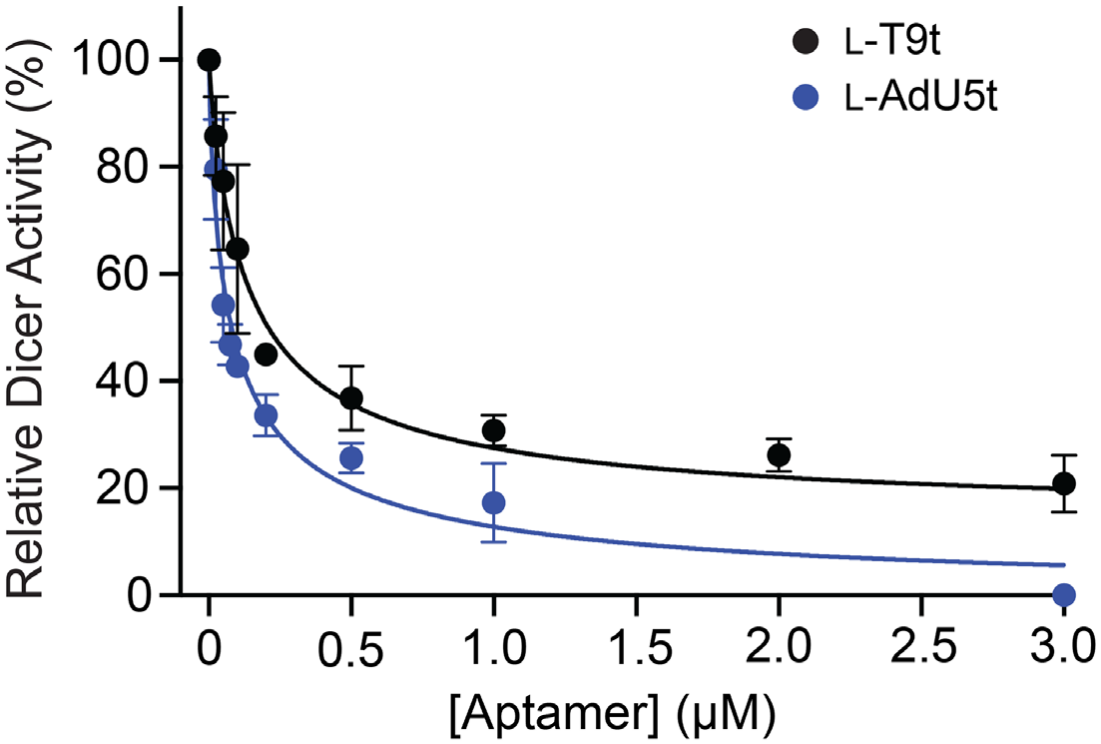
Cross-chiral l-DNA aptamers inhibit Dicer-mediated cleavage of pre-miR-155. Percent Dicer activity (relative to a no l-aptamer control) was plotted as a function of l-aptamer concentration. Each data point represents the mean ± S.D. (*n* = 3). The data were fit to a four-parameter sigmoidal dose-response model and IC_50_ values were determined.

During microRNA biogenesis, the distal stem-loop domain of pre-miRs is excised by the ribonuclease Dicer prior to being loaded onto an Argonuate protein, where it is eventually used to guide sequence-specific silencing of target mRNAs (49). Previous studies have shown that Dicer-catalyzed cleavage of specific pre-miRs can be blocked using reagents that bind the corresponding distal stem-loop domain (50–53), and this strategy is currently being pursued for therapeutic purposes (54). Binding to the loop domain of pre-miRs by our previously reported cross-chiral l-RNA aptamers also inhibited Dicer. Therefore, we predicted that l-T9t and l-AdU5t would be effective at inhibiting Dicer-mediated cleavage of pre-miR-155. To test this, d-pre-miR-155 was incubated in the presence of human Dicer protein, together with various concentrations of either l-T9t or l-AdU5t. We observed a concentration-dependent inhibition of pre-miR-155 processing for both aptamers (Figure 6). The IC_50_ values calculated for l-T9t and l-AdU5t were 139 and 80 nM, respectively, which are in good agreement with their corresponding *K*_d_ values (Figure 2B and C). As a control for non-specific inhibition of Dicer, we found that neither d-T9t nor d-AdU5t inhibited Dicer-mediated cleavage to a significant degree (Supplementary Figure S12), which is consistent with their inability to bind d-pre-miR-155.

## CONCLUSION

In summary, we have demonstrated *in vitro* selection of the first cross-chiral aptamers comprised of l-DNA, thereby establishing the capacity of DNA and RNA molecules of the opposite handedness to form tight and specific interactions with each other. Thus, this work greatly expands our current definition of nucleic acid molecule recognition. Our best l-DNA aptamers had low nM affinities for pre-miR-155, similar to previously identified l-RNA aptamers against the same target. Furthermore, like their RNA counterparts, these l-DNA aptamers bound selectively to the loop domain of pre-miR-155 and inhibited Dicer-mediated processing *in vitro*. Thus, despite prior evidence suggesting that DNA may be less adept than RNA at forming heterochiral interactions (22,25–26), our results clearly show that the lack of a 2′-OH group is not a disadvantage for cross-chiral recognition of RNA. The question of whether DNA or RNA will yield better cross-chiral aptamers in terms of RNA-binding requires further investigation, and may ultimately depend on the identity of the target. Nevertheless, DNA is more stable, easier to produce and less expensive than RNA, making it a more practical choice for the generation of cross-chiral aptamers having applications in biotechnology.

Despite binding to the same site (i.e. nucleotide sequence) on pre-miR-155, the sequences and structures of l-DNA and l-RNA aptamers were found to be distinct from each other. This was somewhat unexpected, as both the aptamer and target are comprised of nucleic acids, providing the opportunity for DNA and RNA aptamers to form similar sequence-specific interactions with the target. Indeed, d-DNA and d-RNA aptamers have been found to bind the same d-RNA target through similar sequence-specific interactions (WC base pairing in this case) (12,55), giving rise to DNA and RNA aptamers with closely related sequences and structures. Even in the absence of WC base pairing between d- and l-oligonucleotides, it is possible that similar sequence-specific interactions could emerge from libraries of both DNA and RNA, although these interactions would likely be more idiosyncratic in nature than WC base pairing. Therefore, the lack of sequence and structural similarities between cross-chiral DNA and RNA aptamers, as well as between T9t and AdU5t, is significant because it rules out this possibility and provides further support for the hypothesis that cross-chiral nucleic acid recognition occurs independent of sequence-specific interaction, instead relying on structure-specific tertiary interactions.

During the course of this study, we explored the potential for cationic modifications (i.e. 5AdU) to facilitate and/or promote cross-chiral interactions between DNA and RNA of the opposite chirality. A previous *in vitro* selection experiment using an RNA library containing the same cationic modification (5-aminoallyluridine) yielded cross-chiral l-RNA aptamers for pre-miR-19a having low nanomolar *K*_d_ value (17). However, a parallel selection experiment using an unmodified RNA library was not carried out, making it difficult to determine what advantages, if any, the cationic modification bestowed. Herein, we found that both modified and unmodified ssDNA libraries readily yielded cross-chiral aptamers against l-pre-miR-155. The *K*_d_ value for the best modified aptamer (AdU5t) was only slightly better (∼2- to 3-fold) than the best unmodified aptamer (T9t) under all conditions tested (Figure 2 and Supplementary Figure S6). Based on these observations, we conclude that the cationic aminoallyl modification did not provide a significant advantage for cross-chiral recognition of pre-miR-155 by DNA. This result was unexpected based on the nature of the target, and is significant because it suggests that the use of cationic modifications may not benefit future cross-chiral aptamer selections. However, we acknowledge these experiments represent a very small sample size and we cannot rule out that different results may be obtained under alternative *in vitro* selection condition and/or for other RNA targets. Importantly, despite originating from the same dsDNA pool, the modified ssDNA library (Lib.AdU) yielded aptamer sequences that were distinct from its native DNA counterpart (Lib.dT) and were dependent on the 5AdU modification for binding. This suggests that the modified DNA pool was able to sample unique structural spaces or distinct interactions with pre-miR-155. Other functional group modifications will likely have different properties that make them more or less amenable toward binding RNA. In contrast to protein targets, for which the influence of unnatural nucleobase modifications on aptamer binding has been studied extensively (30,31), almost nothing is known about what type of modifications are most apt for RNA binding by aptamers. This study now provides a robust platform for screening a diverse set of modified nucleotides in order to identify functional groups capable of enhancing the binding properties of cross-chiral aptamers. It is worth noting that, in general, modified deoxyribose triphosphates and their corresponding phosphoramidites are easier to obtain synthetically than their ribose counterparts due to the absence of the 2′-hydroxyl group, representing another advantage of generating cross-chiral aptamers from libraries of DNA.

Inhibition of miR biogenesis using reagents that target the unique structures of pre-miRs represents a promising therapeutic strategy for a variety of diseases, including cancer (54). Such inhibitors also represent new biochemical tools for interrogating pre-miR function, and can potentially be utilized as affinity reagents for imaging and diagnostic applications. Although several challenges remain, our results demonstrate that cross-chiral l-DNA aptamers have properties well-suited for these purposes, including high affinity and selectivity, nuclease resistance and potent inhibition of Dicer-mediated cleavage. While pre-miRNA-155 served as a convenient proof-of-concept target for this study, it represents one of the most common RNA structures: a hairpin. Therefore, we anticipate that the methods developed herein can be readily applied to the isolation of cross-chiral l-DNA aptamers for many clinically relevant RNAs, including long noncoding RNAs and viral RNA elements.

## Supplementary Material

gkz1236_Supplemental_File

## References

[1] Wan Y., Kertesz M., Spitale R.C., Segal E., Chang H.Y. understanding the transcriptome through RNA structure. Nat. Rev. Genet. 2011; 12:641–655. 21850044 10.1038/nrg3049 PMC3858389

[2] Bernat V., Disney M.D. RNA structures as mediators of neurological diseases and as drug targets. Neuron. 2015; 87:28–46. 26139368 10.1016/j.neuron.2015.06.012 PMC4508199

[3] Croce C.M. Causes and consequences of microRNA dysregulation in cancer. Nat. Rev. Genet. 2009; 10:704–714. 19763153 10.1038/nrg2634 PMC3467096

[4] Engelman A., Cherepanov P. The structural biology of HIV-1: mechanistic and therapeutic insights. Nat. Rev. Microbiol. 2012; 10:279–290. 22421880 10.1038/nrmicro2747 PMC3588166

[5] Freier S.M., Kierzek R., Jaeger J.A., Sugimoto N., Caruthers M.H., Neilson T., Turner D.H. Improved free-energy parameters for predictings of RNA duplex stability. Proc. Natl. Acad. Sci. U.S.A. 1986; 83:9373–9377. 2432595 10.1073/pnas.83.24.9373 PMC387140

[6] Li P.T.X., Vieregg J., Tinoco I.J. How RNA unfolds and refolds. Annu. Rev. Biochem. 2008; 77:77–100. 18518818 10.1146/annurev.biochem.77.061206.174353

[7] Guan L., Disney M.D. Recent advances in developing small molecules targeting RNA. ACS Chem. Biol. 2012; 7:73–86. 22185671 10.1021/cb200447r

[8] Shortridge M.D., Varani G. Structure based approaches for targeting non-coding RNAs with small molecules. Curr. Opin. Struct. Biol. 2015; 30:79–88. 25687935 10.1016/j.sbi.2015.01.008 PMC4416997

[9] Thomas J.R., Hergenrother P.J. Targeting RNA with small molecules. Chem. Rev. 2008; 108:1171–1224. 18361529 10.1021/cr0681546

[10] Ellington A.D., Szostak J.W. *In vitro* selection of RNA molecules that bind specific ligands. Nature. 1990; 346:818–822. 1697402 10.1038/346818a0

[11] Tuerk C., Gold L. Systematic evolution of ligands by exponential enrichment: RNA ligands to bacteriophage T4 DNA polymerase. Science. 1990; 249:505–510. 2200121 10.1126/science.2200121

[12] Duconge F., Toulmé J.-J. In vitro selection identifies key determinants for loop-loop interactions: RNA aptamers selective for the TAR RNA element of HIV-1. RNA. 1999; 5:1605–1614. 10606271 10.1017/s1355838299991318 PMC1369882

[13] Sánchez-Luque F.J., Stich M., Manrubia S., Briones C., Berzal-Herranz A. Efficient HIV-1 inhibition by a 16 nt-long RNA aptamer designed by combining *in vitro* selection and *in**silico* optimization strategies. Sci. Rep. 2014; 4:doi:10.1038/srep06242. 10.1038/srep06242 PMC4150108 25175101

[14] Mayer G., Raddatz M.-S.L., Grunwald J.D., Famulok M. RNA ligands that distinguish metabolite-induced conformations in the TPP riboswitch. Angew. Chem. Int. Ed. 2007; 46:557–560. 10.1002/anie.200603166 17146816

[15] Lünse C., Michlewski G., Hopp C.S., Rentmeister A., Cáceres J.F., Famulok M., Mayer G. An aptamer targeting the apical-loop domain modulates pri-miRNA processing. Angew. Chem. Int. Ed. 2010; 49:4674–4677. 10.1002/anie.200906919 20533473

[16] Scarabino D., Crisari A., Lorenzini S., Williams K., Tocchini-Valentini G.P. tRNA prefers to kiss. EMBO J. 1999; 18:4571–4578. 10449422 10.1093/emboj/18.16.4571 PMC1171531

[17] Kabza A.M., Sczepanski J.T. An L-RNA aptamer with expanded chemical functionality that inhibits microRNA biogenesis. Chem. Bio. Chem. 2017; 18:1824–1827. 10.1002/cbic.201700362 28696509

[18] Sczepanski J.T., Joyce G.F. Specific inhibition of microRNA processing using L-RNA aptamers. J. Am. Chem. Soc. 2015; 137:16032–16037. 26652064 10.1021/jacs.5b06696 PMC4703082

[19] Sczepanski J.T., Joyce G.F. Binding of a structured D-RNA by an L-RNA Aptamer. J. Am. Chem. Soc. 2013; 135:13290–13293. 23977945 10.1021/ja406634g PMC3804424

[20] Garbesi A., Capobianco M.L., Colonna F.P., Tondelli L., Arcamone F., Manzini G., Hilbers C.W., Aelen J.M.E., Blommers M.J.J. L-DNAs as potential antimessenger oligonucleotides: a reassessment. Nucleic Acids Res. 1993; 21:4159–4165. 8414968 10.1093/nar/21.18.4159 PMC310044

[21] Hoehlig K., Bethge L., Klussmann S. Stereospecificity of oligonucleotide interactions revisited: no evidence for heterochiral hybridization and ribozyme/DNAzyme activity. PLoS One. 2015; 10:e0115328. 25679211 10.1371/journal.pone.0115328 PMC4334536

[22] Ashley G.W. Modeling, synthesis, and hybridization properties of (L)-ribonucleic acid. J. Am. Chem. Soc. 1992; 114:9732–9736.

[23] Hauser N.C., Martinez R., Jacob A., Rupp S., Hoheisel J.D., Matysiak S. Utilising the left-helical conformation of L-DNA for analysing different marker types on a single universal microarray platform. Nucleic Acids Res. 2006; 34:5101–5111. 16990248 10.1093/nar/gkl671 PMC1636439

[24] Dunn M.R., Jimenez R.M., Chaput J.C. Analysis of aptamer discovery and technology. Nat. Rev. Chem. 2017; 1:doi:10.1038/s41570-017-0076.

[25] Garbesi A., Capobianco M.L., Colonna F.P., Maflini M., Niccoiai D., Tondelli L. Chirally-modifiedoligonucleotides and the control of gene expression. The case of L-DNAs and-RNAs. Nucleosides Nucleotides. 1998; 17:1275–1287.

[26] D’Alonzo D., Guaragna A., Palumbo G. Exploring the role of chirality in nucleic acid recognition. Chem. Biodivers. 2011; 8:373–413. 21404424 10.1002/cbdv.201000303

[27] Nishioka M., Mizuguchi H., Fujiwara S., Komatsubara S., Kitabayashi M., Uemura H., Takagi M., Imanaka T. Long and accurate PCR with a mixture of KOD DNA polymerase and its exonuclease deficient mutant enzyme. J. Biotechnol. 2001; 88:141–149. 11403848 10.1016/s0168-1656(01)00275-9

[28] Sun D., Hurley L.H. Biochemical techniques for the characterization of G-quadruplex structures: EMSA, DMS footprinting, and DNA polymerase stop assay. Methods Mol. Biol. 2010; 608:65–79. 20012416 10.1007/978-1-59745-363-9_5 PMC2797547

[29] Higgs G., Slack F. The multiple roles of microRNA-155 in oncogenesis. J. Clin. Bioinform. 2013; 3:17. 10.1186/2043-9113-3-17 PMC3849775 24073882

[30] Meek K.N., Rangel A.E., Heemstra J.M. Enhancing aptamer function and stability via *in vitro* selection using modified nucleic acids. Methods. 2016; 106:29–36. 27012179 10.1016/j.ymeth.2016.03.008

[31] Diafa S., Hollenstein M. Generation of aptamers with an expanded chemical repertoire. Molecules. 2015; 20:16643–16671. 26389865 10.3390/molecules200916643 PMC6332006

[32] Rohloff J.C., Gelinas A.D., Jarvis T.C., Ochsner U.A., Schneider D.J., Gold L., Janjic N. Nucleic acid ligands with protein-like side chains: modified aptamers and their use as diagnostic and therapeutic agents. Mol. Therapy. 2014; 3:e201. 10.1038/mtna.2014.49 PMC4217074 25291143

[33] Battersby T.R., Ang D.N., Burgstaller P., Jurczyk S.C., Bowser M.T., Buchanan D.D., Kennedy R.T., Benner S.A. Quantitative analysis of receptors for adenosine nucleotides obtained via in vitro selection from a library incorporating a cationic nucleotide analog. J. Am. Chem. Soc. 1999; 121:9781–9789. 11543572 10.1021/ja9816436

[34] Vaish N.K., Larralde R., Fraley A.W., Szostak J.W., McLaughlin L.W. A novel, modification-dependent ATP-binding aptamer selected from an RNA library incorporating a cationic functionality. Biochemistry. 2003; 42:8842–8851. 12873145 10.1021/bi027354i

[35] Ohsawa K., Kasamatsu T., Nagashima J.I, Hanawa K., Kuwahara M., Ozaki H., Sawai H. Arginine-modified DNA aptamers that show enantioselective recognition of the dicarboxylic acid moiety of glutamic acid. Anal. Sci. 2008; 24:167–172. 18187867 10.2116/analsci.24.167

[36] Vaught J.D., Bock C., Carter J., Fitzwater T., Otis M., Schneider D., Rolando J., Waugh S., Wilcox S.K., Eaton B.E. Expanding the chemistry of DNA for in vitro selection. J. Am. Chem. Soc. 2010; 132:4141–4151. 20201573 10.1021/ja908035g

[37] Sakthivel K., Barbas C.F. III Expanding the potential of DNA for binding and catalysis: highly functionalized dUTP derivatives that are substrates for thermostable DNA polymerases. Angew. Chem. Int. Ed. 1998; 37:2872–2875. 10.1002/(SICI)1521-3773(19981102)37:20<2872::AID-ANIE2872>3.0.CO;2-5 29711104

[38] Lermer L., Roupioz Y., Ting R., Perrin D.M. Toward an RNaseA mimic: a DNAzyme with imidazoles and cationic amines. J. Am. Chem. Soc. 2002; 124:9960–9961. 12188639 10.1021/ja0205075

[39] Asakura J., Robins M.J. Cerium(IV)-mediated halogenation at C-5 of uracil derivatives. J. Org. Chem. 1990; 55:4928–4933.

[40] Milton R.C., Milton S.C., Kent S.B. Total chemical synthesis of a D-enzyme: the enantiomers of HIV-1 protease show reciprocal chiral substrate specificity. Science. 1992; 256:1445–1448. 1604320 10.1126/science.1604320

[41] Zuker M. Mfold web server for nucleic acid folding and hybridization prediction. Nucleic Acids Res. 2003; 31:3406–3415. 12824337 10.1093/nar/gkg595 PMC169194

[42] Tucker W.O., Shum K.T., Tanner J.A. G-quadruplex DNA aptamers and their ligands: structure, function and application. Curr. Pharm. Des. 2012; 18:2014–2026. 22376117 10.2174/138161212799958477

[43] Bhattacharyya D., Mirihana Arachchilage G., Basu S. Metal cations in G-Quadruplex folding and stability. Front. Chem. 2016; 4:38. 27668212 10.3389/fchem.2016.00038 PMC5016522

[44] Vorlíčková M., Kejnovská I., Sagi J., Renčiuk D., Bednářová K., Motlová J., Kypr J. Circular dichroism and guanine quadruplexes. Methods. 2012; 57:64–75. 22450044 10.1016/j.ymeth.2012.03.011

[45] Karsisiotis A.I., Hessari N.M.a., Novellino E., Spada G.P., Randazzo A., Webba da Silva M. Topological characterization of nucleic acid G-quadruplexes by UV absorption and circular dichroism. Angew. Chem. Int. Ed. 2011; 50:10645–10648. 10.1002/anie.201105193 21928459

[46] Deckard C.E., Sczepanski J.T. Polycomb repressive complex 2 binds RNA irrespective of stereochemistry. Chem. Commun. 2018; 54:12061–12064. 10.1039/c8cc07433j 30295686

[47] Seenisamy J., Rezler E.M., Powell T.J., Tye D., Gokhale V., Joshi C.S., Siddiqui-Jain A., Hurley L.H. The dynamic character of the G-quadruplex element in the c-MYC promoter and modification by TMPyP4. J. Am. Chem. Soc. 2004; 126:8702–8709. 15250722 10.1021/ja040022b

[48] Soukup G.A., Breaker R.R. Relationship between internucleotide linkage geometry and the stability of RNA. RNA. 1999; 5:1308–1325. 10573122 10.1017/s1355838299990891 PMC1369853

[49] Wilson R.C., Doudna J.A. Molecular mechanisms of RNA interference. Annu. Rev. Biophys. 2013; 42:217–239. 23654304 10.1146/annurev-biophys-083012-130404 PMC5895182

[50] Pai J., Hyun S., Hyun J.Y., Park S.-H., Kim W.-J., Bae S.-H., Kim N.-K., Yu J., Shin I. Screening of pre-miRNA-155 binding peptides for apoptosis inducing activity using peptide microarrays. J. Am. Chem. Soc. 2016; 138:857–867. 26771315 10.1021/jacs.5b09216

[51] Maiti M., Nauwelaerts K., Herdewijn P. Pre-microRNA binding aminoglycosides and antitumor drugs as inhibitors of Dicer catalyzed microRNA processing. Bioorg. Med. Chem. Lett. 2012; 22:1709–1711. 22257890 10.1016/j.bmcl.2011.12.103

[52] Connelly C.M., Boer R.E., Moon M.H., Gareiss P., Schneekloth J.S. Jr Discovery of inhibitors of microRNA-21 processing using small molecule microarrays. ACS Chem. Biol. 2017; 12:435–443. 27959491 10.1021/acschembio.6b00945 PMC6341489

[53] Chen Y., Yang F., Zubovic L., Pavelitz T., Yang W., Godin K., Walker M., Zheng S., Macchi P., Varani G. Targeted inhibition of oncogenic miR-21 maturation with designed RNA-binding proteins. Nat. Chem. Biol. 2016; 12:717–723. 27428511 10.1038/nchembio.2128 PMC4990487

[54] Di Giorgio A., Duca M. Synthetic small-molecule RNA ligands: future prospects as therapeutic agents. Medchemcomm. 2019; 10:1242–1255. 31534649 10.1039/c9md00195f PMC6748380

[55] Boiziau C., Dausse E., Yurchenko L., Toulme J.J. DNA aptamers selected against the HIV-1 trans-activation-responsive RNA element form RNA-DNA kissing complexes. J. Biol. Chem. 1999; 274:12730–12737. 10212256 10.1074/jbc.274.18.12730

