# Supplementary material for: In vitro selection of l-DNA aptamers that bind a structured d-RNA molecule
Source: Nucleic Acids Res. 2020 Jan 17;48(4):1669–80. doi: 10.1093/nar/gkz1236 (PMC7038948; doi:10.1093/nar/gkz1236)
Supplement: gkz1236_Supplemental_File [file gkz1236_supplemental_file.pdf]

Supporting Information

In Vitro Selection of L-DNA Aptamers that Bind a Structured D-RNA Molecule

Sougata Dey[1] and Jonathan T. Sczepanski[1,*]

[1] Department of Chemistry, Texas A&M University, College Station, Texas, 77843, USA

* To whom correspondence should be addressed. Email: jon.sczepanski@chem.tamu.edu

I. Supplementary Figures.

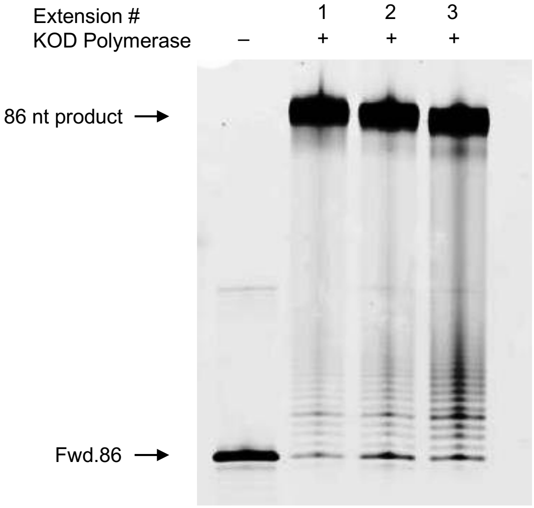

Figure S1. Representative gel (10% denaturing PAGE; 19:1 acrylamide:bisacrylamide) showing multiple extension reactions of 5′-FAM-labeled Fwd.86 (21 nt) on bead-bound Lib.86 (86 nt) using 5-aminoallyl-dUTP and KOD Dash DNA polymerase (lanes 1–3).

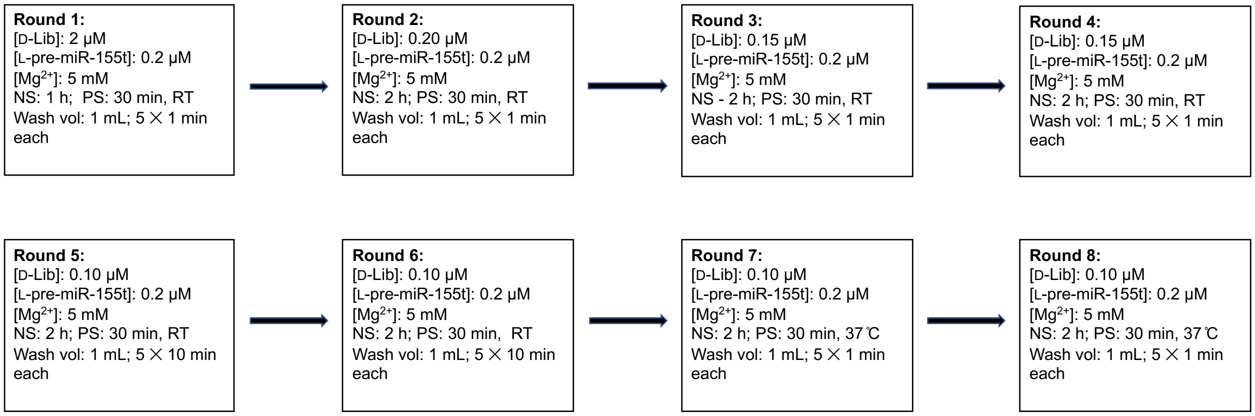

Figure S2. Flow chart detailing conditions used during each round of *in vitro* selection. The amount of time each DNA libraries was incubated with streptavidin-coated beads (negative selection: NS) or L-pre-miR-155t (positive selection: PS) is indicated. The temperature at which the PS was carried out is also indicated. Following immobilization of L-pre-miR-155t to streptavidin-coated beads, non-binding sequences were washed away using the indicated volume of Buffer SB.

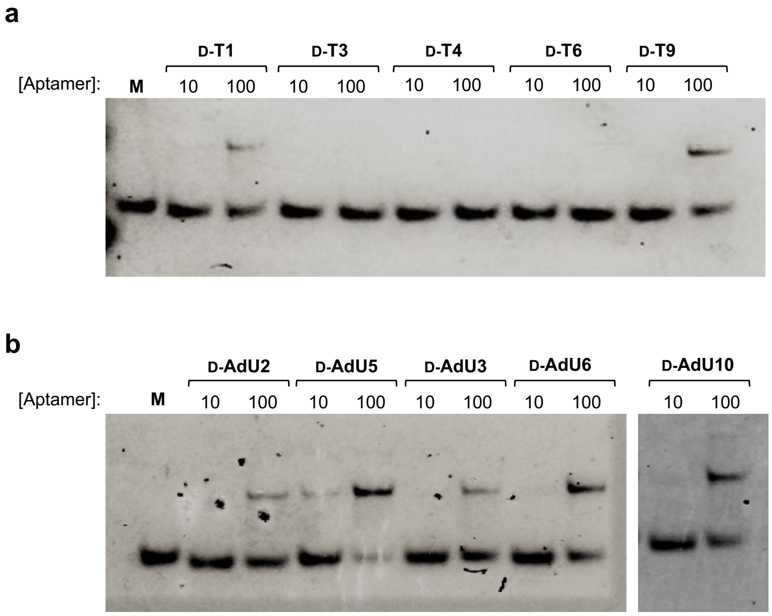

Figure S3. Screening of clones for binding L-pre-miR-155t by EMSA (8% native PAGE; 19:1 acrylamide:bisacrylamide). Each clone represents a unique aptamer sequence isolated from Lib.dT (a) or Lib.AdU (b). All binding reactions contained 5 nM of 5′-FAM-labeled L-pre-miR-155t, either 10 or 100 nM aptamer, 5 mM $MgCl_2$, 50 mM KCl, 20 mM NaCl, 0.1 mg/mL of tRNA, and 25 mM Tris (pH 7.6). M = unbound 5′-FAM-labeled L-pre-miR-155t control.

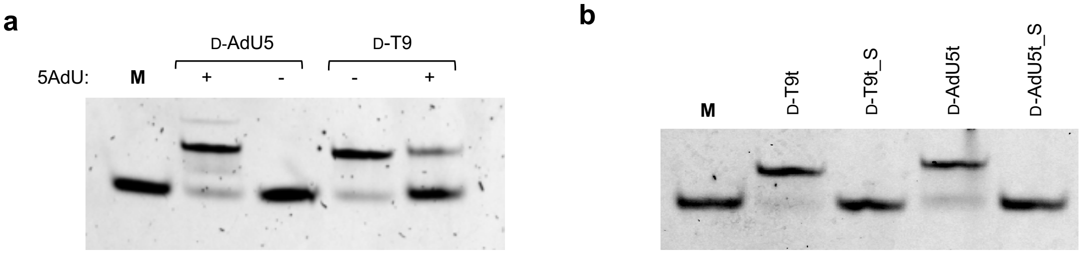

Figure S4. (a) EMSA analysis (8% native PAGE; 19:1 acrylamide:bis-acrylamide) of D-AdU5 and D-T9 containing either 5AdU (+) or dT (-) binding to L-pre-miR-155t. (b) EMSA analysis (8% native PAGE; 19:1 acrylamide:bis-acrylamide) of D-T9t, D-AdU5t, and scrambled variants (D-T9t_S and D-AdU5t_S) binding to L-pre-miR-155t. Sequences of scrambled aptamers D-T9t_S and D-AdU5t_S are listed in Tables S4 and S5, respectively. All binding reactions contained 5 nM of 5′-FAM-labeled L-pre-miR-155t, 3 μM indicated aptamer, 5 mM $MgCl_2$, 50 mM KCl, 20 mM NaCl, 0.1 mg/mL of tRNA, and 25 mM Tris (pH 7.6). M = unbound 5′-FAM-labeled L-pre-miR-155t control.

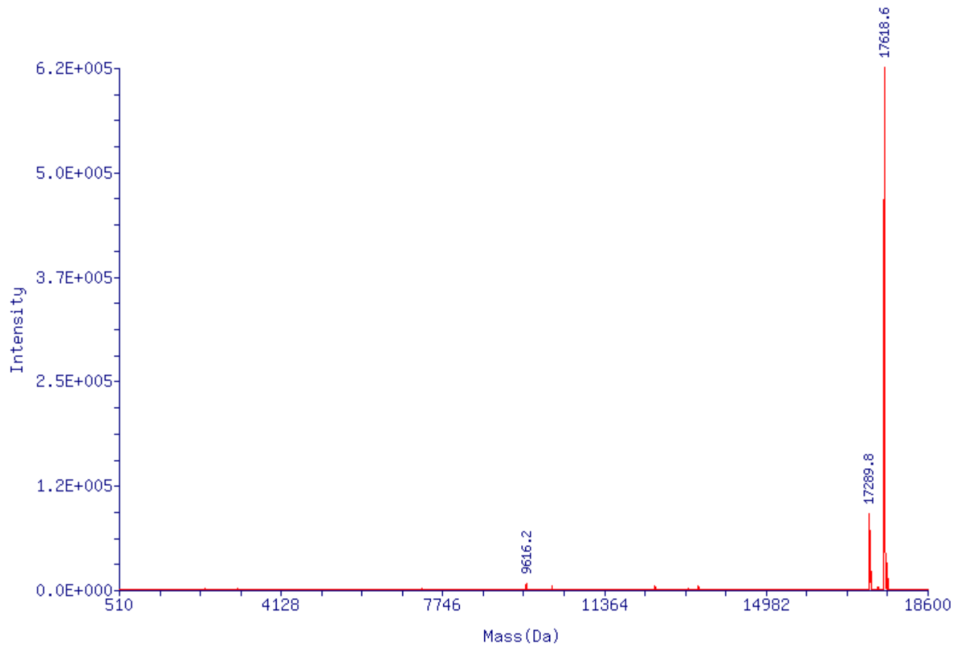

Figure S5. ESI-MS spectrum of synthetic L-T9t. Mass calculated: 17618.4; Mass found: 17618.6.

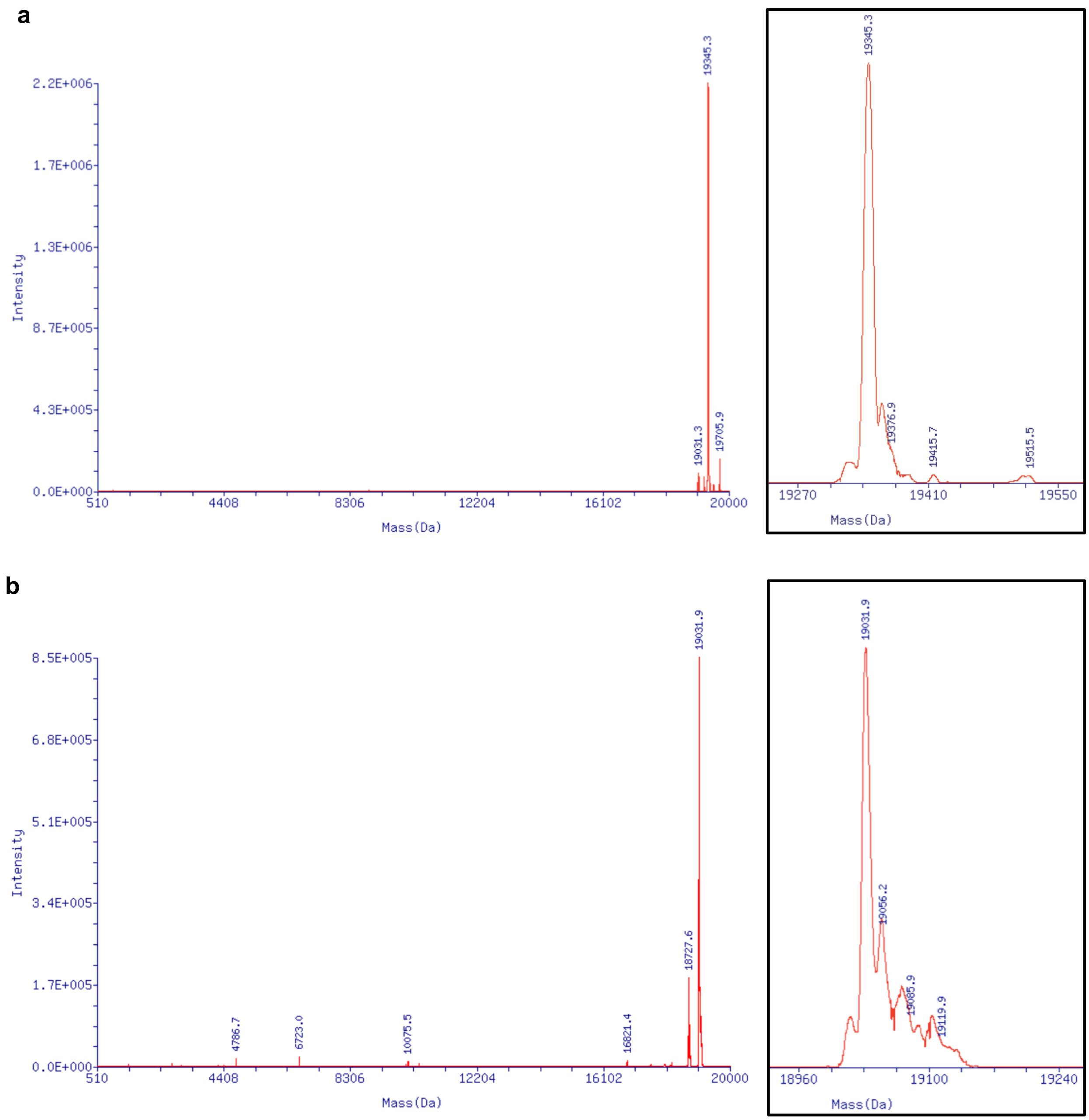

Figure S6. ESI-MS spectra of aptamer AdU5t. (a) ESI-MS of D-AdU5t prepared by enzymatic polymerization using KOD Dash polymerase. Mass calculated: 19345.2; Mass found: 19345.3. (b) ESI-MS of L-AdU5t prepared by solid-phase synthesis. Mass calculated: 19032.3; Mass found: 19031.9. Mass difference of +313.4 (+dA) between D-AdU5t and L-AdU5t is the result of the template independent 3′-dA overhang generated by KOD Dash during polymerization.

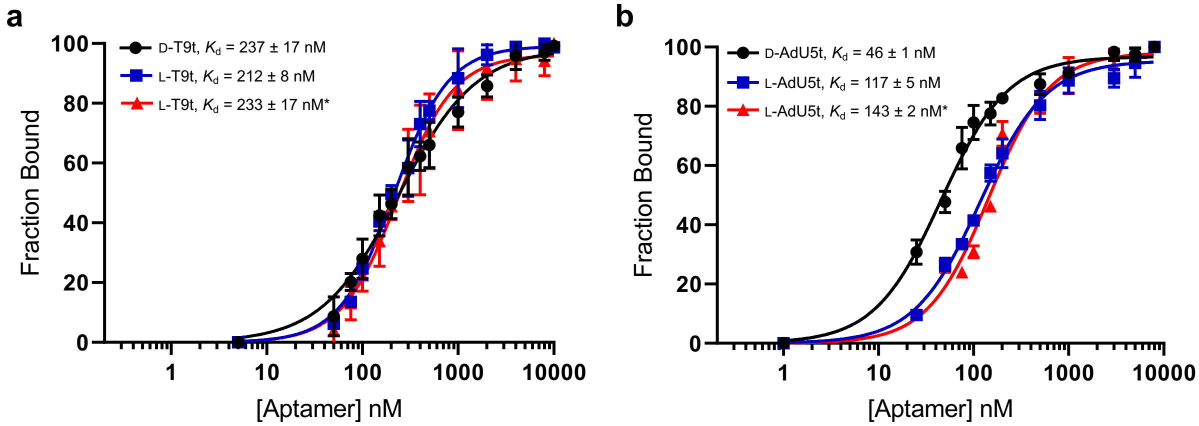

Figure S7. Saturation plots for the binding of cross-chiral DNA aptamers binding pre-miR-155(t) under reduced Mg^{2+} concentration (2 mM). (a) Saturation plots for binding of T9t to either pre-miR-155t or pre-miR-155 (asterisk) having the opposite chirality. Saturation plots for binding of AdU5t to either pre-miR-155t or pre-miR-155 (asterisk) having the opposite chirality. K_d values reported as mean ± S.D. (n = 3). All binding reactions contained either 5 nM of 5′-FAM-labeled L-pre-miR-155t or 1 nM of [5′-^{32}P]-labeled D-pre-miR-155(t), the indicated concentration of aptamer, 2 mM $MgCl_2$, 50 mM KCl, 20 mM NaCl, 0.1 mg/mL of tRNA, and 25 mM Tris (pH 7.6).

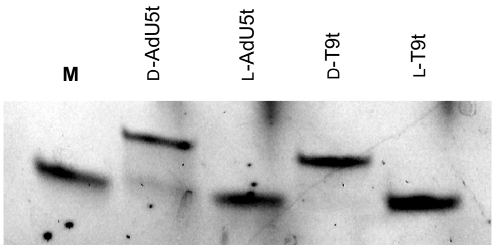

Figure S8. EMSA analysis (8% native PAGE; 19:1 acrylamide:bis-acrylamide) of D- or L-AdU5t and D- or L-T9t binding to L-pre-miR-155t. All binding reactions contained 5 nM of 5′-FAM-labeled L-pre-miR-155t, 3 μM aptamer, 5 mM $MgCl_2$, 50 mM KCl, 20 mM NaCl, 0.1 mg/mL of tRNA, and 25 mM Tris (pH 7.6). M = unbound 5′-FAM-labeled L-pre-miR-155t control.

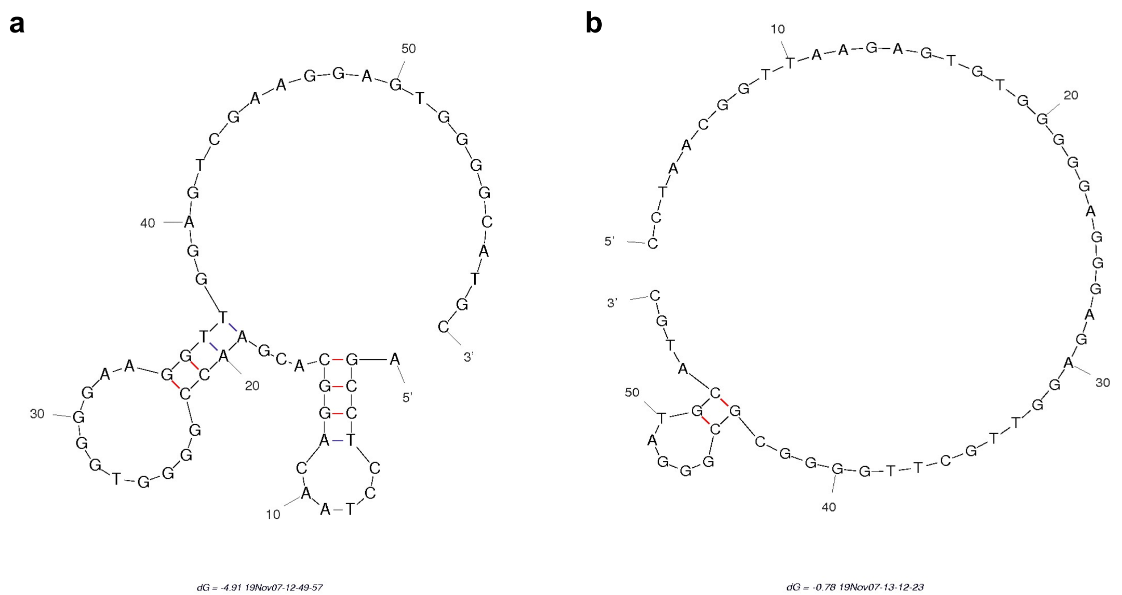

Figure S9. Predicted secondary structures of D-AdU5t (a) and D-T9t (b) using Mfold.

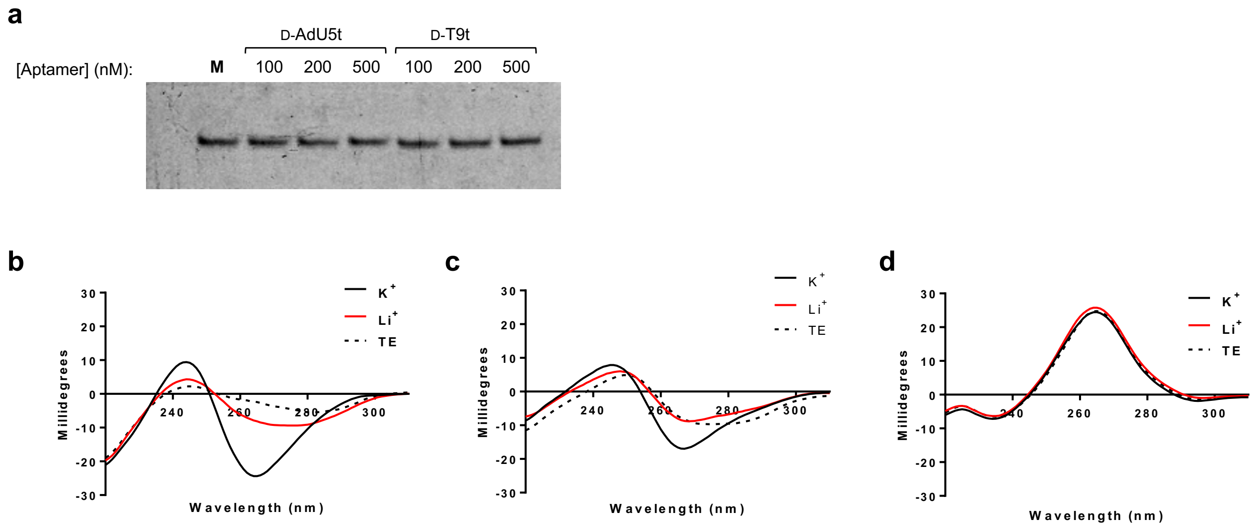

Figure S10. (a) EMSA analysis (8% native PAGE; 19:1 acrylamide:bis-acrylamide) of D-AdU5t and D-T9t binding to L-pre-miR-155t in the absence of K^+. All binding reactions contained 5 nM of 5′-FAM-labeled L-pre-miR-155t, 3 μM aptamer, 5 mM $MgCl_2$, 50 mM LiCl, 20 mM NaCl, 0.1 mg/mL of tRNA, and 25 mM TRIS (pH 7.6). M = unbound 5′-FAM-labeled L-pre-miR-155t control. (b,c,d) Circular dichroism (CD) spectra of L-AdU5t (b), L-T9t (c) and D-RNA aptamiR 155.2 (d) (1). Buffer contained 20 mM NaCl, 25 mM Tris (pH 7.6) and either 50 mM KCl (K^+, shown in black solid line) or LiCl (Li^+, shown in red solid line). CD spectra recorded under no salt condition was prepared in TE buffer (shown in black dashed line).

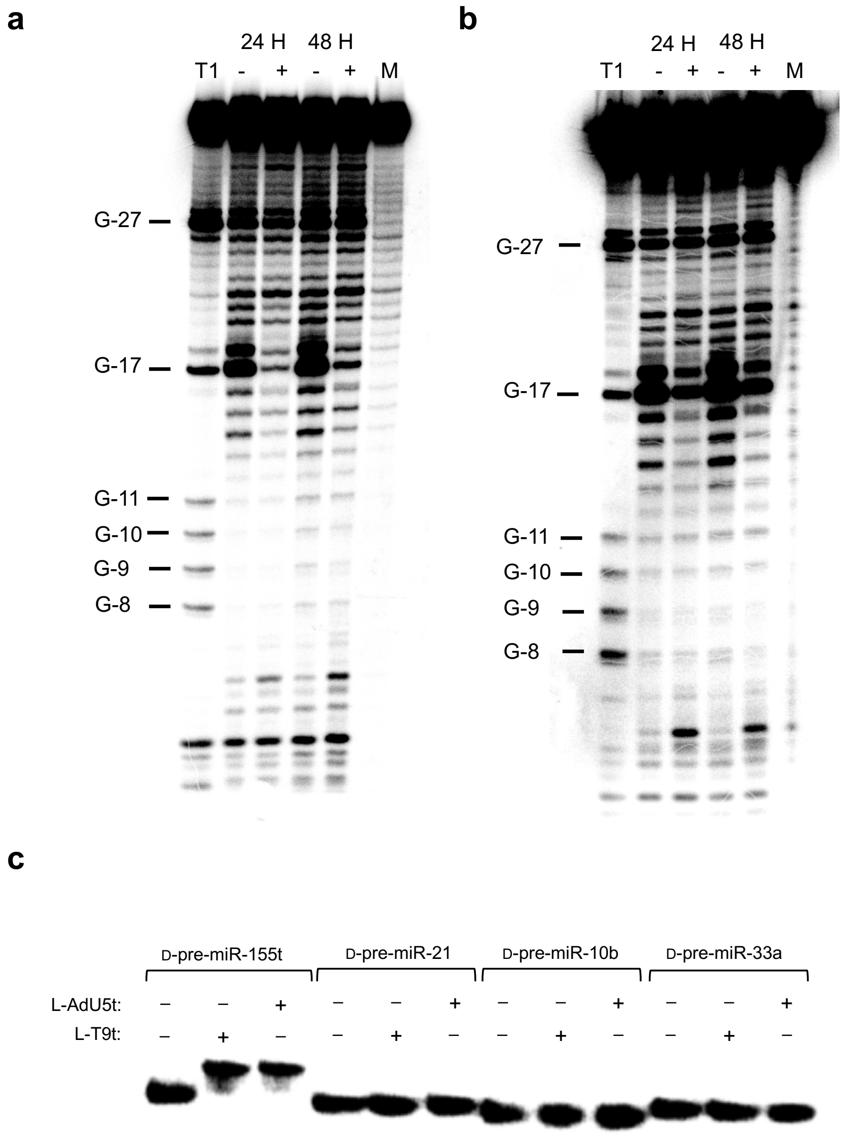

Figure S11. Selective binding of cross-chiral L-DNA aptamers to the distal-loop domain of pre-miR-155. (a,b) Uncropped gel images from Figure 5a in the main text. In-line probing analysis of D-pre-miR-155t in the presence and absence of L-AdU5t (a) or L-T9t (b). All reactions contained 100 nM of 5′-[^{32}P]-labeled D-pre-miR-155t, either none (-) or 20 μM (+) of L-aptamer, 5 mM $MgCl_2$, 50 mM KCl, 20 mM NaCl, and 25 mM Tris (pH 7.6). Unreacted 5′-[^{32}P]-labeled D-pre-miR-155t (M) and material that had been partially digested with ribonuclease T1 (cleaves after G residues) are shown. (c) Discrimination of L-AdU5t and L-T9t against other pre-miR hairpins. All binding reactions contained 5′-[^{32}P]-labeled D-pre-miR, 3 μM aptamer, 5 mM $MgCl_2$, 50 mM KCl, 20 mM NaCl, 0.1 mg/mL of tRNA, and 25 mM Tris (pH 7.6).

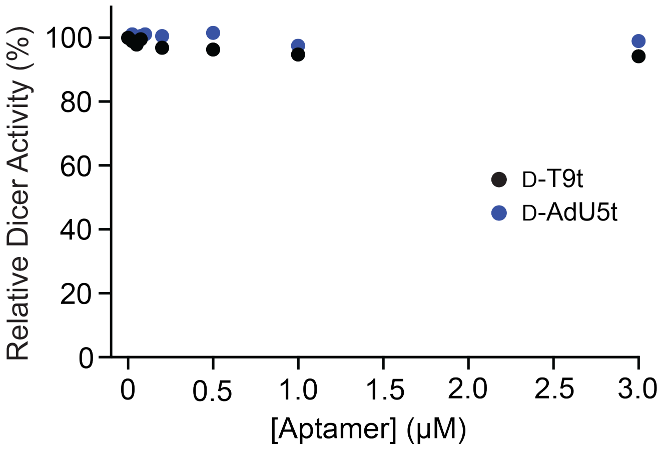

Figure S12. Cross-chiral D-DNA aptamers do not inhibit Dicer-mediated cleavage of D-pre-miR-155. Percent Dicer activity (relative to a no D-aptamer control) was plotted as a function of D-aptamer concentration.

II. Supplementary Tables.

Table S1. DNA sequences associated with library assembly.

**Lib.86**	GCCTGTTGTGAGCCTCCTAAC (**N45**) CATGCTTATTCTTGTCTCCC
**Fwd.86**	GCCTGTTGTGAGCCTCCTAAC
**Rev.86**	5'-/5BiotinTEG/GGGAGACAAGAATAAGCATG

Table S2. Sequences of individual clones (N45 region) isolated from Lib.dT following 8 rounds of *in vitro* selection. The clone having the highest affinity for L-pre-miR-155t is shown in red and the clone found to be enriched in both *in vitro* selection experiments is shown in blue.

**D-T1**	AGGGTGTCAGGTGGTGGGGAGAGGGCATGGACTGGGCGTGGACGG
**D-T2**	AGAGTGTCAGGTGGTGGGGAGAGGGCATGGACTGGGCGTGGACGG
**D-T3**	GGGGGTGGGTGGTCGGGTAATGGAGGAGCGGGCAGTTAGGGTGAA
**D-T4**	TTTACGCACCGATCGCAGGTTAAATTGAGGGGTTATTTCACAGT
**D-T5**	GAGTGTCAGGTGGTGGGGAGAGGACATGGACTGGGCGTGGACGG
**D-T6**	ATATGTGTTCGCATCGGTCGCAGGTAACACATTAACGTCTTTAG
**D-T7**	GGGGGTGGGTGGTCGGGTAATGGAGGAGCGGGCAGTTAGGGTGAA
**D-T8**	AAGTGTCAGGTGGTGGGGAGAGGGCATGGACTGGGCGAGGGACGG
**D-T9**	GGTTAAGAGTGTGGGGGAGGGAGAGGTTGCTTGGGGCGCGGGATG

Table S3. Sequences of individual clones (N45 region) isolated from Lib.AdU following 8 rounds of *in vitro* selection. The clone having the highest affinity for L-pre-miR-155t is shown in red and the clone found to be enriched in both *in vitro* selection experiments is shown in blue.

**D-AdU1**	AGGGGGGTTGTAAGGTTGGAGTCAAAGGGTGGAGTGGGG
**D-AdU2**	AGGGTGTCAGGTGGTGGGGAGAGGGCATGGACTGGGCGTGGACGG
**D-AdU3**	GTTTGGGGAAGTGGATGGAGGCGCAGGGCGGAGTGGGGAGTTGG
**D-AdU4**	AAAGTGTCAGGTGGTGGGGAGAGGGCATGGACTGGGCGTGAACGG
**D-AdU5**	AGGCACGAACCGGGGTGGGGAAGGTTGGAGTCGAAGGAGTGGGG
**D-AdU6**	AGGGTGGAGTGTTAGGGGGTGGCTAGGTTGGCAGGGGAGTGGA
**D-AdU7**	GTGGTGGAGTGCTTAGGGGGTGGCTAGGTTGGCAGGGGAGTGGA
**D-AdU8**	AGAGTGTCAGGTGGTGGGGAGAGGGCATGGACTGGGCGTGGACGG
**D-AdU9**	AGTGTCAGGTGGGGGGGAGAGGGCATGGGAGGGGGGGGGATGG
**D-AdU10**	GGTCGGAGGGGTGGAGGTTAGCATCGGGGTCGGTGGTATGTCGGG

Table S4. Sequences of 5′ and 3′ truncation variants used to determine the minimal binding domain of aptamer D-T9. Residues from fixed primer binding sites are underlined. n.d. = no binding observed.

Aptamer	Truncations	nt	Sequence	Binding Strength
D-T9	-	86	GCCTGTTGTGAGCCTCCTAACGGTTAAGAGTGTGGGGGAGGGAGAGGTTGCTTGGGGCGCGGGATG CATGCTTATTCTTGTCTCCC	+
D-T9t1	5'-10nt	76	AGCCTCCTAACGGTTAAGAGTGTGGGGGAGGGAGAGGTTGCTTGGGGCGCGGGATGCATGCTTATTC TTGTCTCCC	++
D-T9t2	3'-10nt	76	GCCTGTTGTGAGCCTCCTAACGGTTAAGAGTGTGGGGGAGGGAGAGGTTGCTTGGGGCGCGGGATG CATGCTTATT	++
D-T9t3	5'-10nt, 3'-10nt	66	AGCCTCCTAACGGTTAAGAGTGTGGGGGAGGGAGAGGTTGCTTGGGGCGCGGGATGCATGCTTATT	++
**D-T9t**	**5'-15nt, 3'-15nt**	**56**	**CCTAACGGTTAAGAGTGTGGGGGAGGGAGAGGTTGCTTGGGGCGCGGGATGCATGC**	**+++**
D-T9t_S	5'-15nt, 3'-15nt	56	GATGACCCGGGTGTAGGGTTAATCGGGGTGTGTCGGCATGAGAGCGAGAGGTCGGG	n.d
D-T9t4	5'-10nt, 3'-15nt	61	AGCCTCCTAACGGTTAAGAGTGTGGGGGAGGGAGAGGTTGCTTGGGGCGCGGGATGCATGC	+
D-T9t5	5'-10nt, 3'-20nt	56	AGCCTCCTAACGGTTAAGAGTGTGGGGGAGGGAGAGGTTGCTTGGGGCGCGGGATG	n.d.

Table S5. Sequences of 5′ and 3′ truncation variants used to determine the minimal binding domain of aptamer D-AdU5. Residues from fixed primer binding sites are underlined and 5AdU residues are highlighted in blue. n.d. = no binding observed.

Aptamer	Truncations	nt	Sequence	Binding Strength
D-AdU5	-	85	GCCTGTTGTGAGCCTCCTAACAGGCACGAACCGGGGTGGGGAAGGTTGGAGTCGAAGGAGTGGGGCAT GCTTATTCTTGTCTCCC	++
D-AdU5t1	5'-5nt	80	TTGTGAGCCTCCTAACAGGCACGAACCGGGGTGGGGAAGGTTGGAGTCGAAGGAGTGGGGCATGCTTAT TCTTGTCTCCC	++
D-AdU5t2	5'-10nt	75	AGCCTCCTAACAGGCACGAACCGGGGTGGGGAAGGTTGGAGTCGAAGGAGTGGGGCATGCTTATTCTTG TCTCCC	+
D-AdU5t3	3'-5nt	80	GCCTGTTGTGAGCCTCCTAACAGGCACGAACCGGGGTGGGGAAGGTTGGAGTCGAAGGAGTGGGGCAT GCTTATTCTTGT	+
D-AdU5t4	5'-10nt	75	GCCTGTTGTGAGCCTCCTAACAGGCACGAACCGGGGTGGGGAAGGTTGGAGTCGAAGGAGTGGGGCAT GCTTATT	+++
D-AdU5t5	5'-5nt, 3'-5nt	75	TTGTGAGCCTCCTAACAGGCACGAACCGGGGTGGGGAAGGTTGGAGTCGAAGGAGTGGGGCATGCTTAT TCTTGT	+++
D-AdU5t6	5'-5nt, 3'-10nt	70	TTGTGAGCCTCCTAACAGGCACGAACCGGGGTGGGGAAGGTTGGAGTCGAAGGAGTGGGGCATGCTTAT T	+++
D-AdU5t7	5'-5nt, 3'-15nt	65	TTGTGAGCCTCCTAACAGGCACGAACCGGGGTGGGGAAGGTTGGAGTCGAAGGAGTGGGGCATGC	+++
D-AdU5t8	5'-15nt	70	CCTAACAGGCACGAACCGGGGTGGGGAAGGTTGGAGTCGAAGGAGTGGGGCATGCTTATTCTTGTCTCC C	+++
**D-AdU5t**	**5'-10nt, 3'-15nt**	**60**	**AGCCTCCTAACAGGCACGAACCGGGGTGGGGAAGGTTGGAGTCGAAGGAGTGGGGCATGC**	**++++**
D-AdU5t_S	5'-10nt, 3'-15nt	60	CCGTCCGGCAGGAAGGCCACAAAATGAAAGCGCGTGGGATGGGTCCGTGATTGGGGGAGG	n.d.
D-AdU5t9	5'-5nt, 3'-20nt	60	TTGTGAGCCTCCTAACAGGCACGAACCGGGGTGGGGAAGGTTGGAGTCGAAGGAGTGGGGC	+
D-AdU5t10	5'-5nt, 3'-25nt	55	TTGTGAGCCTCCTAACAGGCACGAACCGGGGTGGGGAAGGTTGGAGTCGAAGGAGT	n.d.
D-AdU5t11	5'-15nt, 3'-15nt	55	CCTAACAGGCACGAACCGGGGTGGGGAAGGTTGGAGTCGAAGGAGTGGGGCATGC	+++

Table S6. Oligonucleotides used to prepare D-pre-miR-155, D-pre-miR-155t and related mutants via cross-extension reaction.

**D-pre-miR-155t**	5' - TTCTAATACGACTCACTATAGGTGATAGGGGTTTTTGCCTCCAACTGACTCCTACACC - 3'<br>5' - GGTGTAGGAGTCAGTTGGAGGCAAAAACCCCTATCACCTATAGTGAGTCGTATTAGAA -3'
**D-pre-miR-155**	5' - TTCTAATACGACTCACTATAGCTGTTAATGCTAATCGTGATAGGGGTTTTTGCCTCCAAC - 3'<br>5' - CTGTTAATGCTAATATGTAGGAGTCAGTTGGAGGCAAAAACCCCT - 3'
**D-pre-miR-155 M1**	5' - TTCTAATACGACTCACTATAGGTGATAGGGGTTTTG GCC - 3'<br>5' - GGTGTAGGAGTCAGTCAGAGGCCAAAACCCCTATCACCT - 3'
**D-pre-miR-155 M2**	5' - TTCTAATACGACTCACTATAGGTGATAGGGGTTTAACCC - 3'<br>5' - GGTGTAGGAGTCAGAACGAGGGTTAAACCCCTATCACCT - 3'
**D-pre-miR-155 M3**	5' - TTCTAATACGACTCACTATAGGTGATAGGGGTTTTTGGA - 3'<br>5' - GGTGTAGGAGTCAGTTGTTTCCAAAAACCCCTATCACCT - 3'
**D-pre-miR-155 M4**	5' - TTCTAATACGACTCACTATAGGTGATAGGGGTTTTTGCC - 3'<br>5' - GGTGTAGGAGTCAGTTGAGGCAAAAACCCCTATCACCTA - 3'
**D-pre-miR-155 M5**	5' - TTCTAATACGACTCACTATAGGTGATAGGGGTTGTTGCC - 3'<br>5' - GGTGTAGGAGTCGTTGGAGGCAACAACCCCTATCACCTA - 3'

III. Supplementary Methods: Synthesis of L-5-aminoallyl-dU CEP (L-5AdU-CEP).

General

The moisture sensitive reactions were carried out under dry Argon. CH_2Cl_2 was distilled over CaH_2. THF was dried and distilled over sodium/benzophenone prior to use. For thin layer chromatography (TLC) Silica gel 60, F254 plates (Merck); Flash chromatography (fc): Silica gel 60, 40–64mm (Merck) were used. NMR spectra were recorded with Bruker 500-MR (500 MHz for ^{1}H NMR spectra, 126 MHz for ^{13}C NMR spectra) or Bruker 400-MR (400 MHz for ^{1}H NMR spectra, 101 MHz for ^{13}C NMR spectra); ppm related to tetramethylsilane and measured referring to $CHCl_3$ (d= 7.26 ppm for ^{1}H NMR and d= 77.16 ppm for^{13}C NMR) and CHD_2OD (d= 3.31 ppm for ^{1}H NMR and d= 49.0 ppm for ^{13}C NMR); coupling constants are given with 0.5 Hz resolution; assignment of ^{13}C and ^{1}H NMR signals was supported by 2-D NMR techniques where necessary.

The synthesis of the D-5AdU-CEP has already been described (2-4). However, the procedures are given here in detail to show some important modifications we made during the course of synthesis. Additionally, the synthesis of unnatural enantiomer L-5AdU-CEP is not yet reported.

Scheme 1. Synthesis of L-5AdU-CEP.

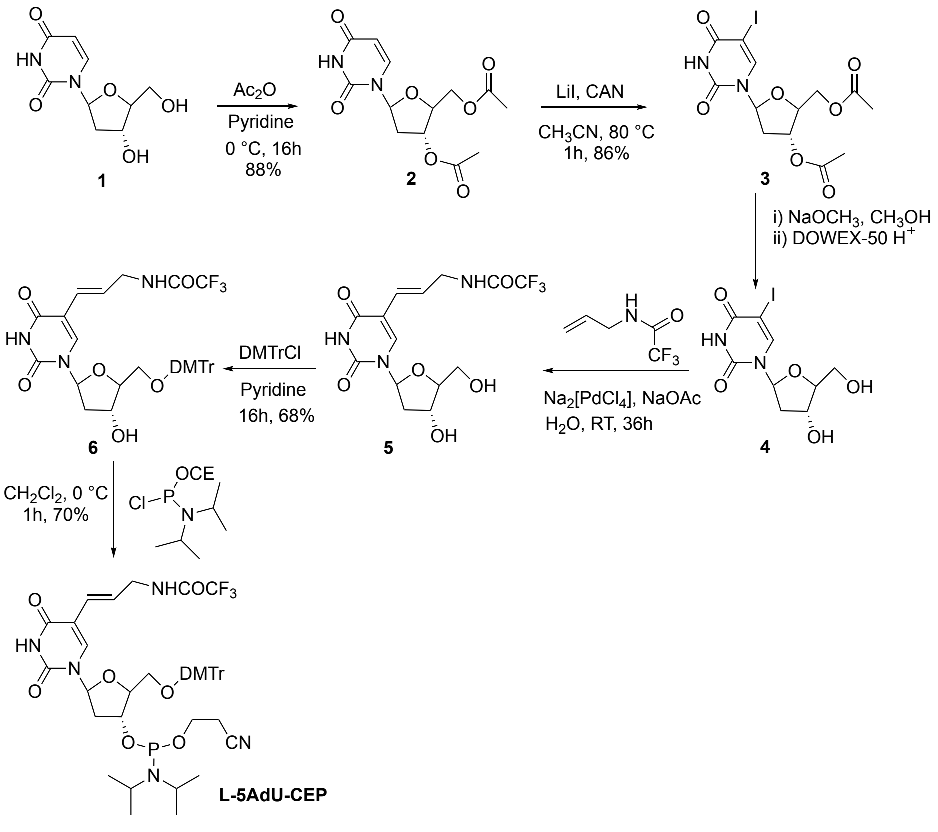

Experimental procedures

((3R)-3-acetoxy-5-(5-iodo-2,4-dioxo-3,4-dihydropyrimidin-1(2H)-yl)tetrahydrofuran-2-yl)methyl acetate (3)

A solution of 500 mg of L-deoxyuridine in 2.5 mL dry pyridine was cooled to 0 ºC in an ovendried two neck flask under Ar. 1.7 mL of Ac_2O was slowly added dropwise over 1 hr and the reaction mixture was stirred for 16 h at RT. After completion of the reaction (monitored by TLC) pyridine was evaporated and the white residue was co-evaporated twice with toluene to obtain acetyl derivative **2** as white foam. A mixture of **2** (500 mg, 1.6 mmol), LiI (260 mg, 1.92 mmol), CAN (1.8 gm, 3.2 mmol) in 25 mL dry MeCN was heated to 80 ºC for 1 h. After the reaction was completed (monitored by TLC, $CHCl_3$: Acetone 4:1), the solvent was evaporated and the residue was partitioned between cold ethyl acetate (40 mL), brine (20 mL) and 5% $NaHSO_3$ (10 mL). The organic layer was further washed with 10 mL cold 5% $NaHSO_3$, 10 mL H_2O and 10 mL brine. The organic layer was dried over $MgSO_4$, concentrated in vacuum and purified by recrystalization from hot ethanol to provide acetyl derivative **3** as white needles (600 mg, 1.38 mmol, 86 %). ^{1}H NMR (400 MHz, $CDCl_3$) δ (ppm): 2.12 (s, 3H, Ac-CH_3), 2.21 (s, 3H, Ac-CH_3), 2.17 (m, 1H, H-2'), 2.54 (ddd, *J* = 14.3, 5.6 Hz, 1.8 Hz, 1H, H-2′), 4.30 (m, 1H, H-3′), 4.34 (dd, *J* = 12.3, 2.8 Hz, 1 H, H-5′), 4.41 (dd, *J* = 12.3, 2.9 Hz, 1H, H-5′), 5.23 (m, 1H, 4-H′), 6.28 (dd, *J* = 8.8, 5.6 Hz, 1H, H-1′), 7.97 (s, 1H, 6-H), 8.54 (broad-s, 1H, N-H).^{13}C NMR (101 MHz, CDCl3) δ (ppm): 21.0 (Ac-CH_3), 21.3 (Ac-CH_3), 31.1 (C-2′), 63.9 (C-5′), 74.2 (C-3'), 77.3 (^{q}C-I), 82.9 (C-4'), 85.6 (C-1′), 143.8 (C-6), 149.7 (^{q}*C*O), 159.5 (^{q}*C*O), 170.3 (Ac-^{q}*C*O), 170.4 (Ac-^{q}*C*O). HR-MS: Calculated ($C_{13}H_{15}IN_2O_7Na+$) 460.9816, found 460.9804.

1-((4R)-4-hydroxy-5-(hydroxymethyl)tetrahydrofuran-2-yl)-5-iodopyrimidine-2,4(1H,3H)-dione (4)

The acetylated L-iododeoxyuridine **3** (500 mg, 1.14 mmol) was stirred with 0.1 M $NaOCH_3$ / CH_3OH (13 mL) for 1 h at RT. Reaction progress was monitored by TLC (EtOAc/^{i}PrOH/H_2O - 4:1:2). 3 mL of H_2O was added and the mixture was neutralized by Dowex 50W-X8 (H^+) resin.(5) The resin was filtered and vigorously washed with warm 50 % aqueous CH_3OH. The combined filtrate was evaporated to dryness and further dried by co-evaporation with 1:1:2 mixture of EtOH/EtOAc/Toluene. The crude product was recrystallized from hot H_2O to provide L-iododeoxyuridine **4** (255 mg, 0.72 mmol, 63 %) as white needles. ^{1}H NMR (400 MHz, DMSO-d_6) δ (ppm): 2.11 (m, 2H, H-2′), 3.59 (m, 2H, H-5′), 3.79 (q, *J* = 3.1 Hz, 1H, H-4′), 4.23 (m, 1H,

H-3′), 5.14 (t, *J* = 4.5 Hz, 1H, 5′-O*H*), 5.23 (d, *J* = 3.9 Hz, 1H, 3′-O*H*), 6.09 (t, *J* = 6.5 Hz, 1H, H-1′), 8.39 (s, 1H, 6-H), 11.68 (broad-s, 1H, N-H).^{13}C NMR (101 MHz, DMSO-d_6) δ (ppm): 40.1 (C-2′), 60.8 (C-5′), 69.7 (C-3′), 70.1 (^{q}C-I), 84.6 (C-1′), 87.5 (C-4′), 145.0 (C-6), 150.2 (^{q}*C*O) 160.9 (1C, ^{q}*C*O). HR-MS: Calculated ($C_9H_{11}IN_2O_5Na^+$) 376.9605, found 376.9598.

2,2,2-trifluoro-N-((E)-3-(1-((4R)-4-hydroxy-5-(hydroxymethyl)tetrahydrofuran-2-yl)-2,4-dioxo-1,2,3,4-tetrahydropyrimidin-5-yl)allyl)acetamide (5) and

N-((E)-3-(1-((4R)-5-((bis(4-methoxyphenyl)(phenyl)methoxy)methyl)-4-hydroxytetrahydrofuran-2-yl)-2,4-dioxo-1,2,3,4-tetrahydropyrimidin-5-yl)allyl)-2,2,2-trifluoroacetamide (6)

A suspension of L-5-iodo-2'-deoxyuridine **4** (2.4 g, 6.7 mmol) in sodium acetate buffer (0.1M, pH 5.2) was treated with N-allyltrifluroacetylamide (9.4g, 61mmol) followed by a solution of sodium tetrachloropalladate (1.8 g in 2.5mL water). The mixture was stirred at room temperature for 36 h and then filtered through celite. The filtrate was concentrated and extracted three times with ethyl acetate (3x20 mL). The combined organic layers were dried over anhydrous $MgSO_4$. The solvent was evaporated to dryness and the product was purified by column chromatography on silica gel with ethyl acetate as an eluent which provided **5** as a mixture along with starting material iododeoxyuridine **4**. ^{1}H NMR (400 MHz, CD_3OD) δ (ppm): 2.17 (m, 2H, H-2′), 3.75-3.60 (m, 2H, H-5′), 3.80 (m, 3H, H-allyl, H-4'), 4.32 (m, 1H, H-3'), 6.16 (m, 2H, H-vinyl, H-1'), 6.45 (m, 1H), 8.11 (broad-s, 1H, H-6).

A solution of aminoallyl derivative **5** (300 mg, 0.79 mmol) in anhydrous pyridine (3 mL) was treated with 4,4’-dimethoxytrityl chloride (407 mg, 1.2 mmol) at 0 ºC and the solution was stirred overnight at RT. The next day, an additional 200 mg of 4,4′-dimethoxytrityl chloride was added and allowed to stir at RT for another 4 h until >90 % of the starting material was consumed (monitored by TLC). 2 mL of CH_3OH was added and the solution was dried under vacuum. The yellow foam was dissolved in 10 mL CH_2Cl_2 and washed with 5% $NaHCO_3$. The aqueous layer was extracted with CH_2Cl_2 (20 mL) and combined organic layer was dried over $MgSO_4$. The solution was filtered, concentrated in vacuum and purified by flash column chromatography (1-5% CH_2Cl_2 / CH_3OH with 0.1 % Et_3N). The DMTr derivative **6** was obtained as white foam (368 mg, 0.5 mmol, 68%). ^{1}H NMR (400 MHz, $CDCl_3$) δ (ppm): 2.33 - 2.40 (m, 1H, H-2′), 2.46 (ddd, *J* = 13.5, 5.9 Hz, 3.1 Hz, 1H, H-2′), 3.36 (d, *J* = 10.7, 2.7 Hz, 1H, H-5′), 3.45 - 3.48 (m, 1H, C*H*$_2$NH), 3.51 (dd, *J* = 10.6, 2.7 Hz, 1 H, H-5′), 3.66 - 3.73 (m, 1H, C*H*$_2$NH), 3.80 (s, 6H, DMTr-

OCH_3), 4.09 (q, *J* = 2.7 Hz, 1H, H-3′), 4.62 (dt, *J* = 5.6, 2.9 Hz, 1H, H-4′), 5.35 (d, *J* = 15.7 Hz, 1H, H-vinyl), 6.31 (dt, *J* = 15.7, 6.9 Hz, 1H, H-allyl), 6.42 (dd, *J* = 7.0, 6.1 Hz, 1H, H-1’), 6.43 - 6.47 (broad-s, 1H, N-H), 6.80 - 6.88 (m, 4H, Arom-CH), 7.22 - 7.40 (m, 9H, Arom-CH), 7.86 (s, 1H, 6-H), 8.80 (broad-s, 1H, N-H). ^{13}C NMR (101 MHz, $CDCl_3$) δ (ppm): 41.9 (C-2′), 42.6 (CH_2NH), 55.4 (OCH_3), 55.5 (1C, OCH_3), 63.5 (C-5′), 72.3 (C-3′), 85.2 (C-4′), 86.9 (C-1′), 87.4 (1C, *C*-DMTr), 111.4 (q, CF_3), 113.6 (Arom-OCH_3), 125.3 (C-5), 128.9 (C-vinyl), 129.1 (C-allyl), 127.4, 128.3, 128.4, 130.4, 130.5, 135.6, 135.7 (Arom), 144.0 (C-6), 149.4 (*C*OCF_3), 158.9 (^{q}*C*O) 161.8 (^{q}*C*O). HR-MS: Calculated ($C_{35}H_{34}F_3N_3O_8Na^+$) 704.2190, found 704.2174.

(3R)-2-((bis(4-methoxyphenyl)(phenyl)methoxy)methyl)-5-(2,4-dioxo-5-((E)-3-(2,2,2-trifluoroacetamido)prop-1-en-1-yl)-3,4-dihydropyrimidin-1(2H)-yl)tetrahydrofuran-3-yl-(2-cyanoethyl) diisopropylphosphoramidite (L-5AdU-CEP)

A solution of DMTr-derivative **6** (200 mg, 0.29 mmol) in dry CH_2Cl_2 was treated with N,N-diisopropylethylamine (145 µL, 0.85 mmol) in a septum sealed oven dried flask under Ar at 0 ℃. Then chloro-N,N-diisopropylaminomethoxyphosphine (116 µL, 0.42 mmol) was added dropwise and stirred at RT for 30 min. After completion of the reaction (monitored by TLC, 5 % CH_2Cl_2 / CH_3OH) the desired phosphoramidite **L-5AdU-CEP** was purified by flash column chromatography under Ar atmosphere (gradient of 20%-50% dry EtOAc-Hexane, 0.1 % Et_3N). A diastereomeric mixture was obtained as white foam (185 mg, 70 %). ^{1}H NMR (400 MHz, $CDCl_3$) δ (ppm): 1.15 - 1.20 (m, 12H, CH_3), 2.37 - 2.40 (m, 1H, H-2′), 2.52 - 2.60 (m, 1H, H-2′), 2.61 - 2.66 (m, 2H, N-C*H*), 3.31 - 3.36 (m, 2H, CH_2NH), 3.45 – 3.48 (m, 1H, H-5′), 3.52 - 3.66 (m, 5H, H-5′, OCH_2-CH_2), 3.79 - 3.80 (s, 6H, DMTr-OCH_3), 4.15 – 4.20 (m, 1H, H-3′), 4.67 – 4.72 (m, 1H, H-4′), 5.28 - 5.31 (m, 1H, H-vinyl), 6.25 - 6.31 (m, 2H, H-allyl, N-H), 6.38 - 6.43 (m, 1H, H-1′), 6.80 - 6.86 (m, 4H, Arom-CH), 7.14 - 7.18 (m, 2H, Arom-CH), 7.27 - 7.42 (m, 7H, Arom), 7.86 (s, 0.5H, 6-H), 7.89 (s, 0.5H, 6-H), 8.15 (broad-s, 1H, N-H). ^{31}P NMR (400 MHz, $CDCl_3$) δ (ppm): 149.25, 149.56, impurity 15.29 (<5%). HR-MS: Calculated ($C_{44}H_{51}F_3N_5O_9Na^+$) 904.3269, found 904.3245.

NMR Spectra (^{1}H, ^{13}C and ^{31}P)

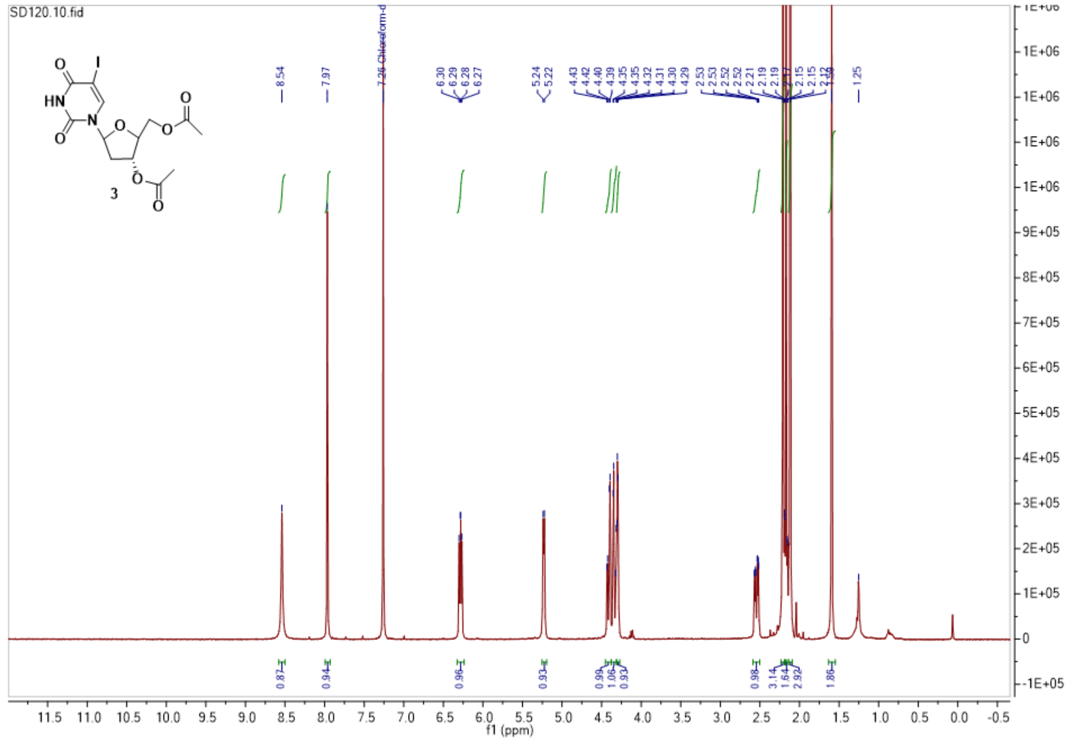

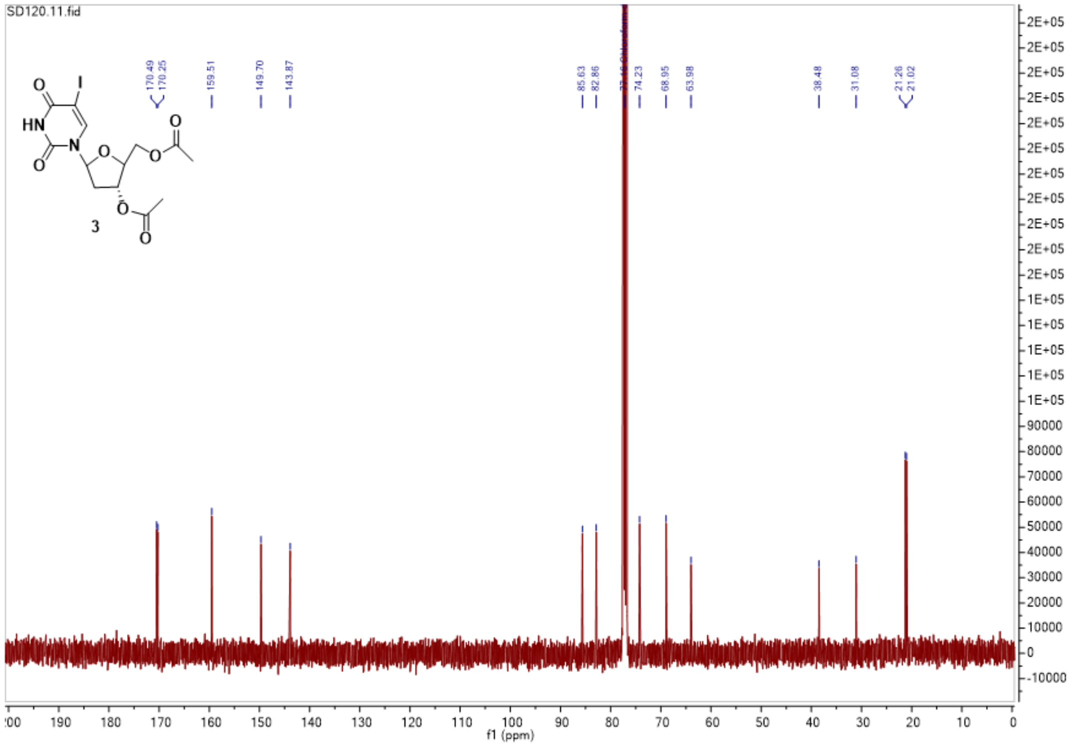

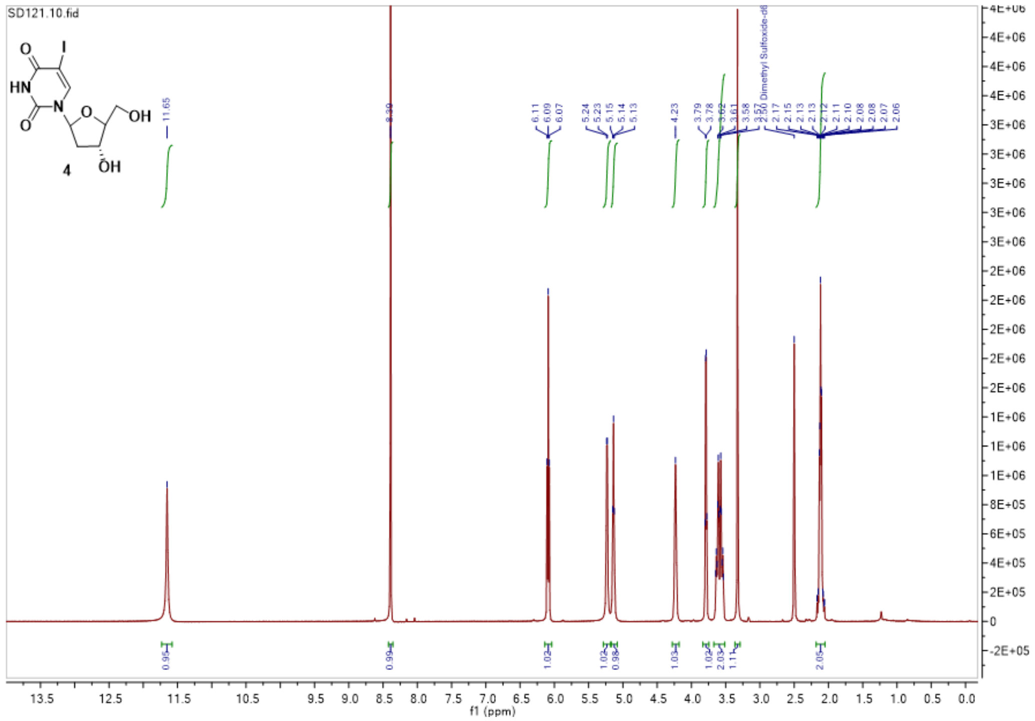
SD121.10.fid
4
f1 (ppm)

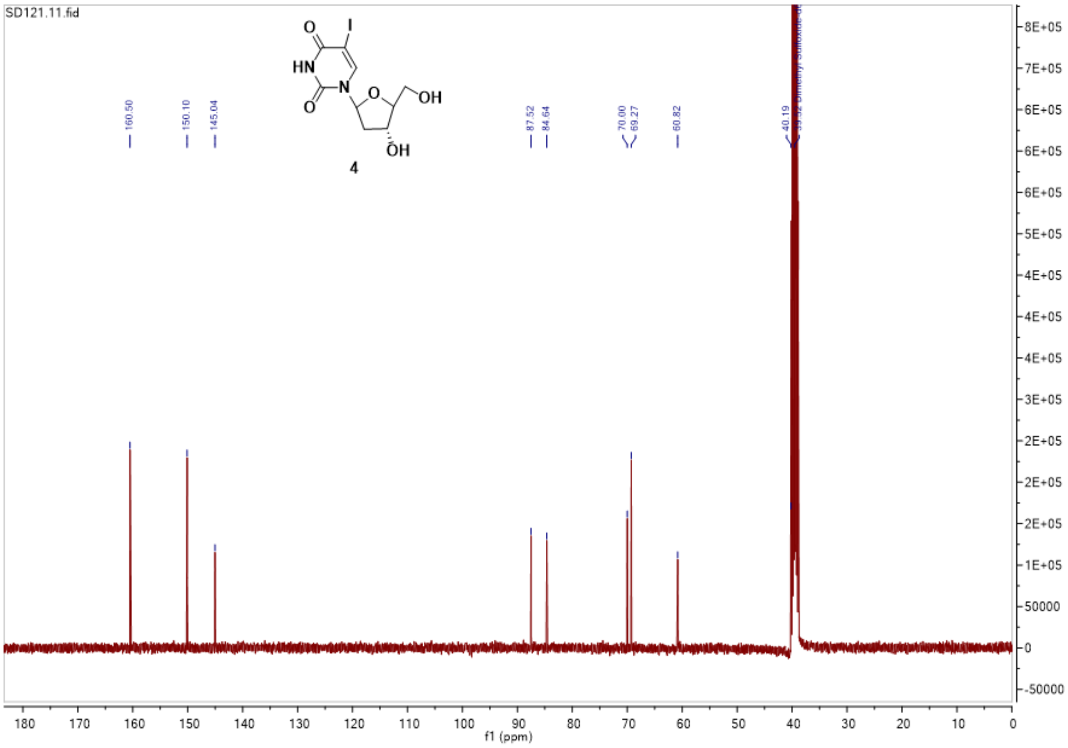
SD121.11.fid
4
160.50
150.10
145.04
87.52
84.64
70.00
69.27
60.82
f1 (ppm)

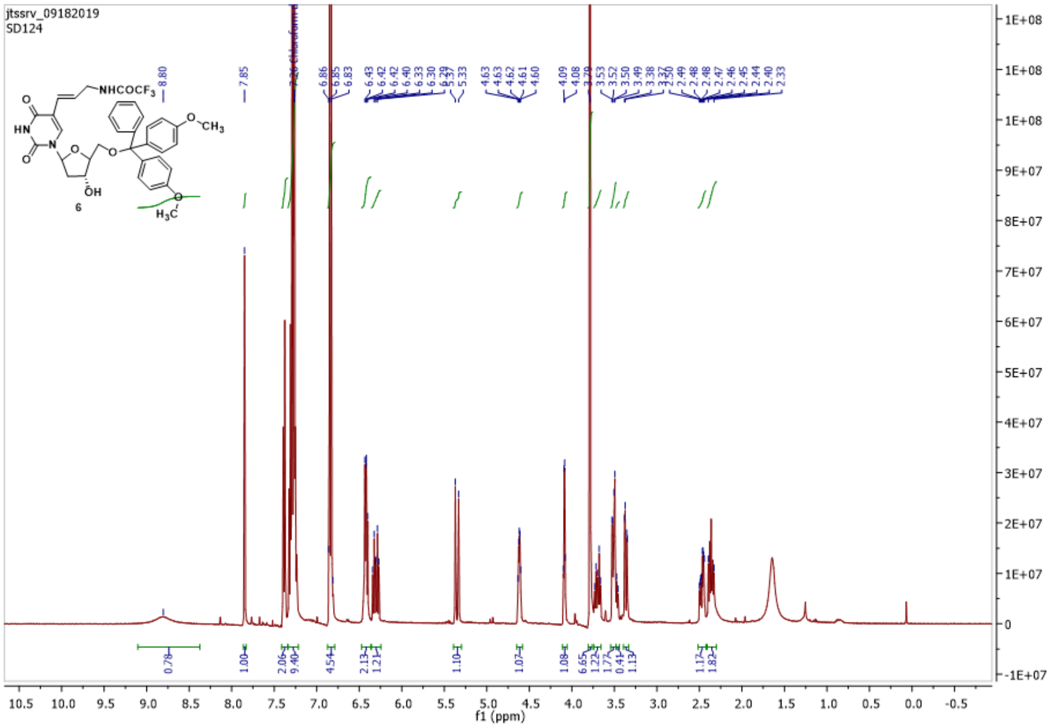
jtssrv_09182019
SD124
NHCOCF3
HN
OH
O-CH3
H3C
6
f1 (ppm)

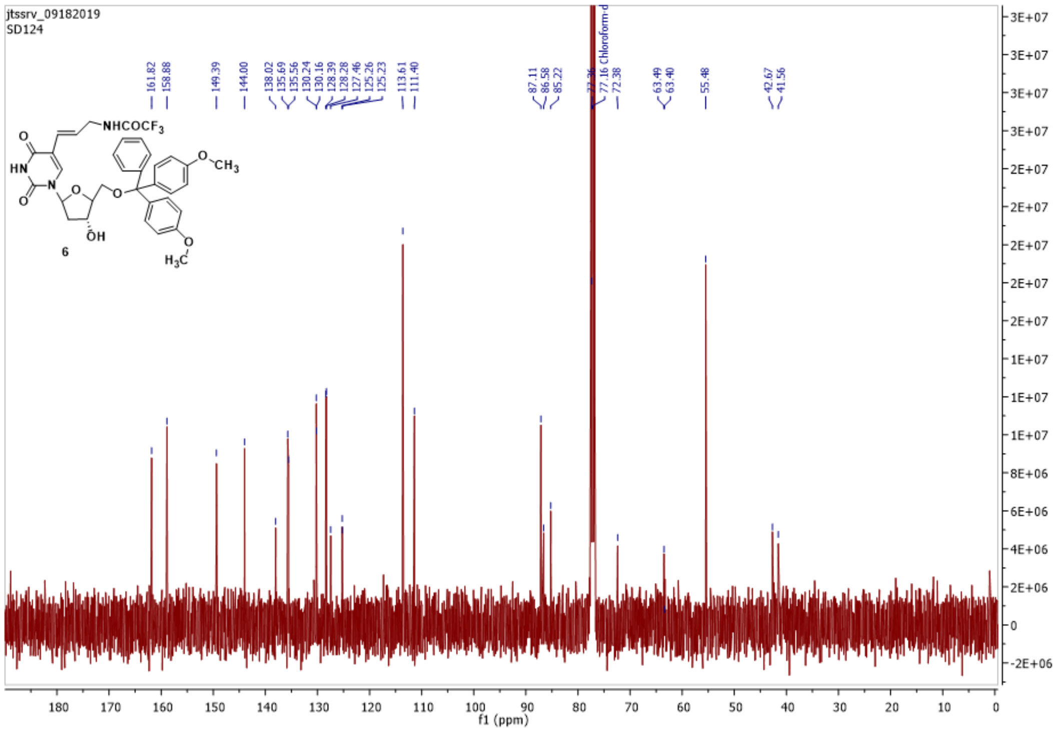
jtssrv_09182019
SD124
NHCOCF3
HN
OH
O-CH3
H3C
6
77.16 Chloroform-d
f1 (ppm)

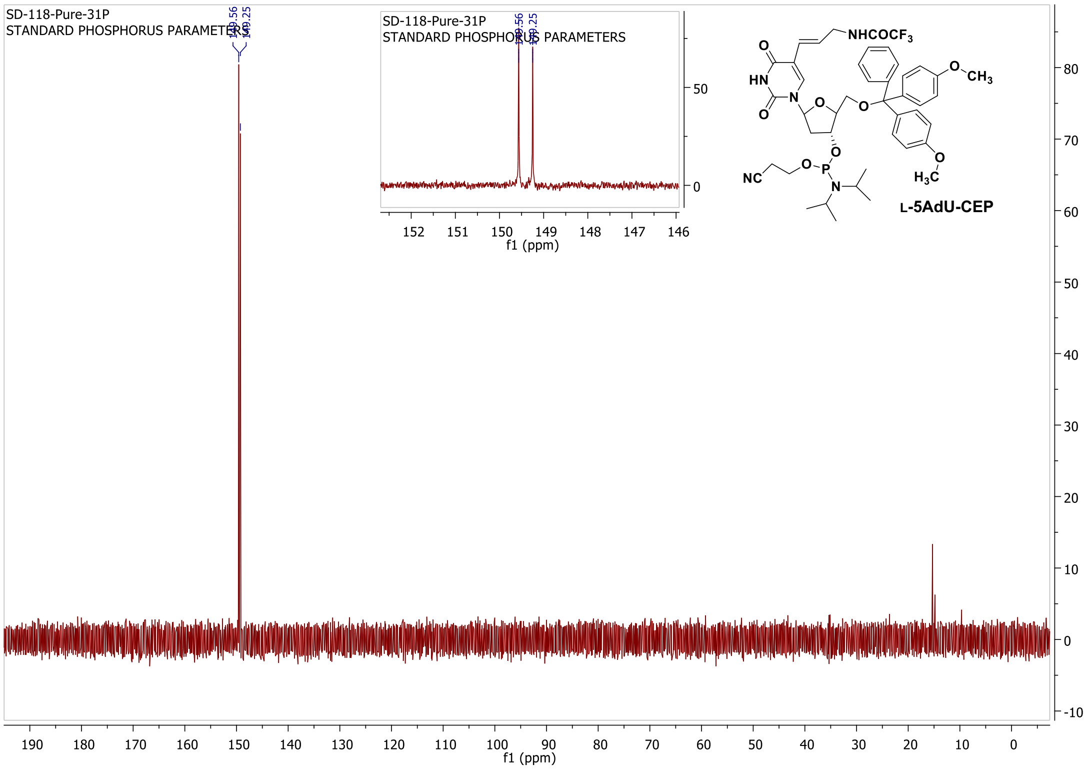
SD-118-Pure-31P
STANDARD PHOSPHORUS PARAMETERS
149.56
149.25
NHCOCF3
HN
NC
L-5AdU-CEP
f1 (ppm)

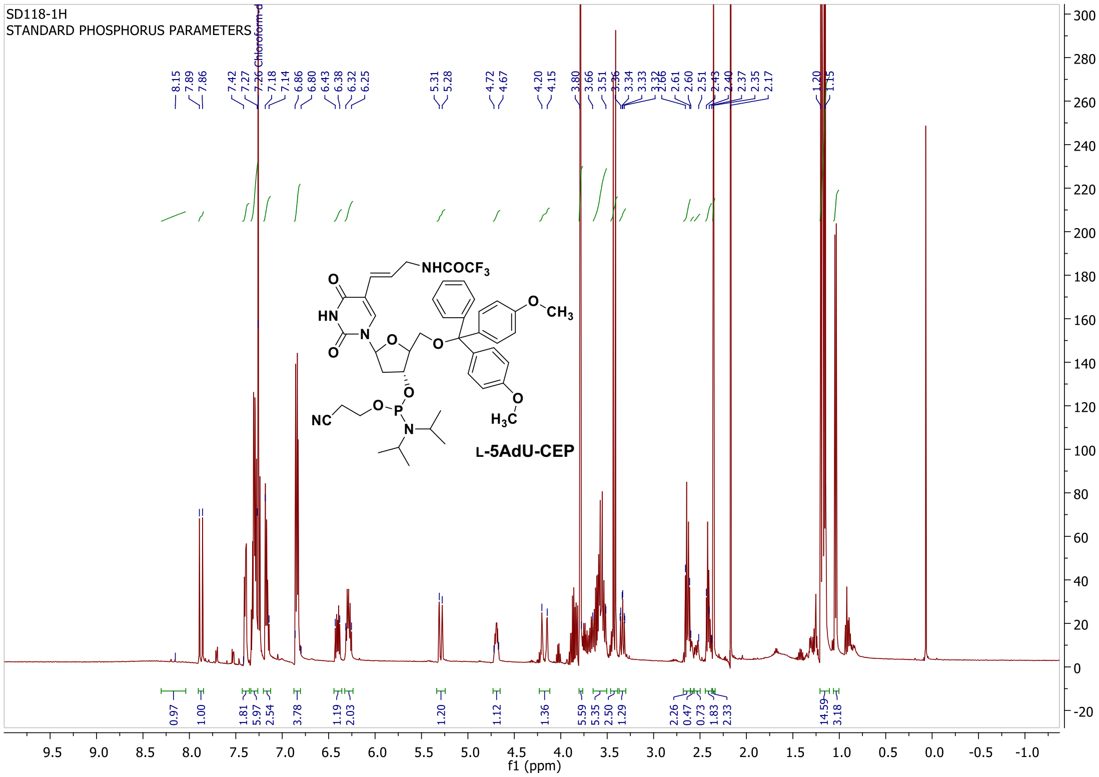
SD118-1H
STANDARD PHOSPHORUS PARAMETERS
7.26 Chloroform-d
NHCOCF3
HN
NC
L-5AdU-CEP
f1 (ppm)

IV. References.

1. Sczepanski, J.T. and Joyce, G.F. (2015) Specific Inhibition of MicroRNA Processing Using L-RNA Aptamers. *J. Am. Chem. Soc.*, **137**, 16032-16037.
2. Cook, A.F., Vuocolo, E. and Brakel, C.L. (1988) Synthesis and hybridization of a series of biotinylated oligonucleotides. *Nucleic Acids Res.*, **16**, 4077-4095.
3. Lermer, L., Roupioz, Y., Ting, R. and Perrin, D.M. (2002) Toward an RNaseA Mimic: A DNAzyme with Imidazoles and Cationic Amines. *J. Am. Chem. Soc.*, **124**, 9960-9961.
4. Sakthivel, K. and Barbas Iii, C.F. (1998) Expanding the Potential of DNA for Binding and Catalysis: Highly Functionalized dUTP Derivatives That Are Substrates for Thermostable DNA Polymerases. *Angew. Chem. Int. Ed.*, **37**, 2872-2875.
5. Asakura, J. and Robins, M.J. (1990) Cerium(IV)-mediated halogenation at C-5 of uracil derivatives. *J. Org. Chem.*, **55**, 4928-4933.
